# The interactions between a small molecule and G-quadruplexes are visualized by fluorescence lifetime imaging microscopy

**DOI:** 10.1038/ncomms9178

**Published:** 2015-09-09

**Authors:** Arun Shivalingam, M. Angeles Izquierdo, Alix Le Marois, Aurimas Vyšniauskas, Klaus Suhling, Marina K. Kuimova, Ramon Vilar

**Affiliations:** 1 Department of Chemistry, Imperial College London, South Kensington, London SW7 2AZ, UK; 2 Department of Physics, King's College London, Strand, London WC2R 2LS, UK

## Abstract

Guanine-rich oligonucleotides can fold into quadruple-stranded helical structures known as G-quadruplexes. Mounting experimental evidence has gathered suggesting that these non-canonical nucleic acid structures form *in vivo* and play essential biological roles. However, to date, there are no small-molecule optical probes to image G-quadruplexes in live cells. Herein, we report the design and development of a small fluorescent molecule, which can be used as an optical probe for G-quadruplexes. We demonstrate that the fluorescence lifetime of this new probe changes considerably upon interaction with different nucleic acid topologies. Specifically, longer fluorescence lifetimes are observed *in vitro* for G-quadruplexes than for double- and single-stranded nucleic acids. Cellular studies confirm that this molecule is cell permeable, has low cytotoxicity and localizes primarily in the cell nucleus. Furthermore, using fluorescence lifetime imaging microscopy, live-cell imaging suggests that the probe can be used to study the interaction of small molecules with G-quadruplexes *in vivo*.

## Abstract (web summary)

Guanine-rich oligonucleotides can fold into secondary structures known as G-quadruplexes that are proposed to have various biological roles. Here, Shivalingam *et al.* develop a cell-permeable, low-toxicity probe that can be used to probe interactions between G-quadruplexes and small molecules *in vivo*.

## Graphical abstract

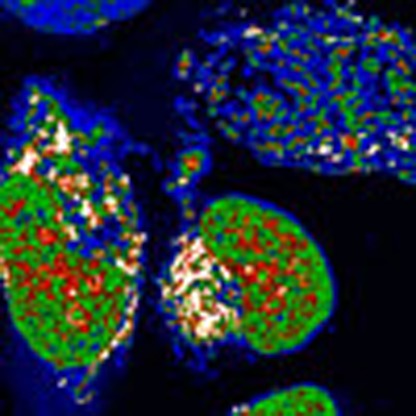

The dynamic nature of DNA enables a range of non-canonical structures to potentially form *in vivo*. G-quadruplexes, in particular, have received increasing attention having been implicated in biologically important roles such as genomic instability, telomerase dysfunction and the regulation of gene expression^1^,^2^,^3^. Yet there still remains considerable debate as to whether these structures form *in vivo*; given that G-quadruplexes arise from non-covalent interactions, isolating them in their native form from live cells is a very challenging task. As a result, many studies have focused on fixed cells where the structural dynamics of nucleic acids can be more readily controlled. Encouragingly, in this context, G-quadruplexes have been visualized using antibodies^4^,^5^,^6^. However, questions remain as to whether these observations are the result of artefacts from chromatin fixation^7^,^8^, or if the high affinity of the antibodies induce G-quadruplex formation. Moreover, dynamic relationships between G-quadruplex formation and its cellular consequences are precluded from these studies. Of these points, cell-permeable small-molecule optical probes would provide an ideal means of monitoring these structures in live cells and in real-time overcoming the limitation of fixed cell artefacts^9^,^10^. Furthermore, such probes would provide invaluable insight into the criteria required for ‘drugability' of G-quadruplexes. While a number of optical probes for G-quadruplexes have been reported over the past few years, to date none of these has been convincingly used to image them in live cells^11^,^12^,^13^,^14^.

High levels of selectivity for G-quadruplexes over duplex DNA have been achieved *in vitro* by designing molecules that have the appropriate shape and size to fit on top of the G-tetrad^15^. Typical ligand features include large aromatic surfaces for end-on *π*–*π* stacking with G-tetrads, a central positive charge to favourably polarize the aromatic system and steric bulk that prevents intercalation into double-stranded (ds) DNA. However, establishing the extent to which these features translate to target engagement in live cells is not a trivial process^16^,^17^. Hence, the development of probes that can incorporate these features while possessing photophysical properties that can be monitored *in vivo* could provide crucial information regarding quadruplex–ligand interactions.

Highly stable planarized triarylmethyl carbocations named trianguleniums provide an ideal platform for such a probe (Fig. 1). These compounds have suitable structural features to interact with nucleic acids, they can be functionalized^18^,^19^,^20^,^21^ and, more importantly, they have intrinsic fluorescence that arises from the aromatic core^22^,^23^ Yet, surprisingly, no DNA binding studies have been reported for variants of the first reported triangulenium, namely **TOTA**^24^,^25^. This is probably due to the limited number of non-alkyl modifications reported to date together with the modest affinity of **TOTA** for DNA (*K*_a_∼10^4^). Furthermore, as is typical of small-molecule optical probes, nucleic acids quench the emission of **TOTA**. Nonetheless, it was envisaged that, first, subtle alteration of the electronic and in turn redox properties of the aromatic core would enable a fluorescence ‘turn-on' system to be obtained; and that, second, the affinity of triangulenium core for nucleic acids (in particular G-quadruplexes) could be significantly enhanced by the addition of appropriately placed substituents to the core.

Herein we present the synthesis and photophysical characterization of new triangulenium derivatives **ADOTA-M** and **DAOTA-M2** (Fig. 1). We demonstrate a ‘turn-on' fluorescence enhancement for **DAOTA-M2** in the presence of nucleic acids as well as significant dependence of its fluorescence lifetime on nucleic acid topology using a wide range of *in vitro* DNA and RNA models. After showing that it is possible to detect G-quadruplexes even in the presence of other competing nucleic acid topologies, we also report fluorescence lifetime imaging microscopy (FLIM) studies of this probe in live cells. Our data show that this probe has a great potential to study the interaction between small molecules and G-quadruplexes in live cells.

## Results

### Synthesis of optical probes

**Figure 1:**
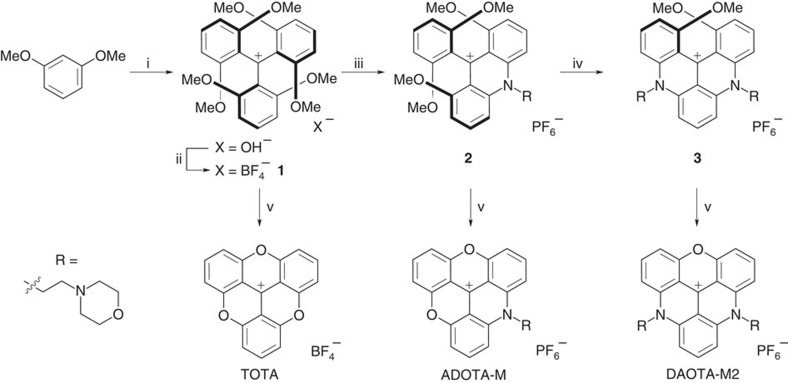
Synthetic route to trianguleniums. (i) ^*n*^BuLi, TEMED, Et_2_O, 0 °C to rt then (EtO)_2_CO, 18 h; (ii) HBF_4_·Et_2_O, Et_2_O, rt, 5 min; (iii) RNH_2_, NMP, room temperature, 2 h then NH_4_PF_6 (aq)_; (iv) RNH_2_, NMP, 110 °C, 2 h then NH_4_PF_6(aq)_; (v) Py·HCl, 200 °C, 1 h.

The new compounds **ADOTA-M** and **DAOTA-M2** were prepared following procedures previously reported for analogous mono- and bis-substituted trianguleniums (Fig. 1)^20^. Both compounds show the expected resonances in the ^1^H nuclear magnetic resonance spectra where the integration between the core aromatic protons and the aliphatic substituents are consistent with mono- and bis-substituted trianguleniums for **ADOTA-M** and **DAOTA-M2**, respectively. The formulations were confirmed by mass spectrometry and elemental analyses (Supplementary Methods).

### Photophysical properties of TOTA, ADOTA-M and DAOTA-M2

**Figure 2:**
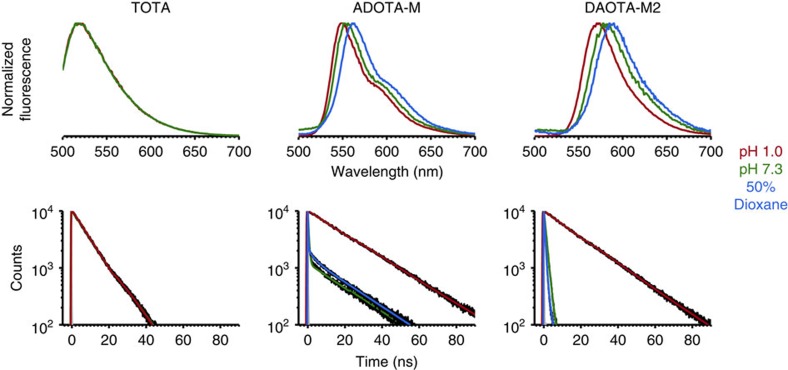
The effect of the local aqueous environment on the fluorescence of **TOTA**, **ADOTA-M** and **DAOTA-M2**. pH 1.0 (red) and 7.3 (green) measurements were recorded in 0.1 M HCl and 10 mM lithium cacodylate buffer containing 100 mM KCl (pH 7.3). Measurements in 50% 1,4-dioxane (blue) were recorded at pH 7.3. For TCSPC traces, raw data and the IRF are coloured black and grey, respectively.

Trianguleniums have interesting photophysical properties, which can be used for sensing and imaging. Yet the incorporation of tertiary amines covalently linked to the fluorophore can significantly alter these properties^26^. Thus, before studying their interaction with nucleic acids, the response of the compounds to changes in the local aqueous environment (for example, oxygen concentration, polarity, viscosity and pH) was investigated and the main changes observed are summarized in Fig. 2 and Table 1. As will be discussed later, this information provides important clues regarding the photophysical changes the probes undergo upon their interaction with nucleic acids.

Under physiological conditions (10 mM lithium cacodylate (pH 7.3) and 100 mM KCl), the quantum yields of **ADOTA-M** and **DAOTA-M2** are 0.042 and 0.032, respectively. Neither variation of ionic strength (0–220 mM KCl) nor deoxygenation of the solutions, have any significant effect upon fluorescence. However, simultaneously decreasing solvent polarity and increasing viscosity by the addition of 1,4-dioxane results in ∼0.3-fold reduction of fluorescence and a red shift in emission maxima.

The time correlated-single photon counting (TCSPC) fluorescence lifetime data show that bi-exponential decays (or tri-exponential in the case of 1,4-dioxane/water mixtures) are necessary to adequately describe the above systems. This suggests that at least two emitting species are present in solution for **ADOTA-M** and **DAOTA-M2**. An excited state equilibrium between the carbocation and carbinol forms of the compounds can be excluded due to the mono-exponential decay of **TOTA**, which has the lowest p*K*_R_^+^ (a measure of carbocation stability) for triangulenium compounds reported to date^27^. As a consequence, protonation of the morpholino group(s) was explored.

Steady-state emission pH titrations reveal the excited p*K*_a_* to be 4.68±0.10 and 4.54±0.13 for **ADOTA-M** and **DAOTA-M2** (±values=s.d., *n*=3; Supplementary Fig. 5), respectively, while **TOTA**, which has no amines, shows no such pH dependence. Comparison of the spectra at pH 7.3 and 1.0 shows that protonation of the amines results in a substantial enhancement of fluorescence (**∼**10–15-fold) and long-lived mono-exponential fluorescence decays (Fig. 2 and Table 1). Furthermore, at pH 7.3, the fluorescence intensity decay of **DAOTA-M2** is not concentration dependent (0.2–50 μM; Supplementary Fig. 6). Therefore, as is well established in the literature^28^, this indicates that the shorter fluorescence lifetime component at pH 7.3 is due to intramolecular photo-induced electron transfer (PET) interaction between a donor amine and excited state acceptor fluorophore.

It is also interesting to note that at pH 7.3, **ADOTA-M** retains a substantial proportion of the long-lived fluorescence component, whereas for **DAOTA-M2** this value is significantly lower. This suggests that in addition to protonation, conformational changes at the nanosecond time scale may be of importance. In the case of **DAOTA-M2**, the number of morpholino groups incorporated in the molecule most likely enhances the probability of PET.

### Interactions of trianguleniums with DNA

Having established the key photophysical properties of these molecules, the effect of different DNA topologies on the steady-state emission of the three trianguleniums under study was investigated. These investigations showed that irrespective of topology, DNA strongly quenches the emission of **TOTA** (as previously reported^24^) and **ADOTA-M**. Interestingly, this was not the case with **DAOTA-M2**, which displayed fluorescence enhancement on the addition of DNA. This enhancement is unlikely to be associated with changes in protonation since a red shift (1–4 nm) in emission was observed on binding to DNA rather than the blue shift associated to protonation (Fig. 2 and Table 1). It is also unlikely to be associated with the local environment inside DNA—which has low polarity—as the photophysics in low polarity 1,4-dioxane/water mixtures (see above) suggests that fluorescence should be quenched. As a consequence of this unexpected DNA binding-dependent behaviour, the potential of the compound to be a ‘turn-on' probe for different DNA topologies was explored.

First, emission titrations were conducted to determine the affinity of **DAOTA-M2** to single-stranded (ss) DNA (ss17), dsDNA (CT-DNA and ds17) and G-quadruplex DNA containing two, three or four guanine tetrads (that is, TBA, myc2345 and PDGF-A, respectively). All binding isotherms, calculated association constants and errors are shown in Supplementary Fig. 7 (as well as absorption titrations that were used to determine compound–DNA stoichiometries). These studies show that **DAOTA-M2** binds with reasonable affinity (log *K*_a_ between 5.7 and 6.1) to all the DNA structures under study.

Moreover, while fluorescence enhancement in the presence of G-quadruplex structures is greater than those of other DNA models (G-quadruplex=3.3–4.9; ssDNA/dsDNA=2.0–2.8; initial quantum yield 0.032), these differences by steady-state intensity alone would be insufficient for cellular imaging of DNA topologies. This is owing to possible differences in uptake/intracellular localization altering the effective concentration of the probe and, in turn, the intensity observed. On the other hand, fluorescence lifetimes are concentration independent, and therefore fluorescence lifetime measurements with **DAOTA-M2** have the potential for high-contrast measurements to distinguish between different nucleic acid topologies. To do this, TCSPC-FLIM was used to measure the emission lifetime of **DAOTA-M2** in the presence of a broad range of DNA (dsDNA or ssDNA, 13 models), RNA (dsRNA or ssRNA, 6 models) and G-quadruplex structures (RNA or DNA based, 6 models, see Methods section for comprehensive list of all models).

**Figure 3:**
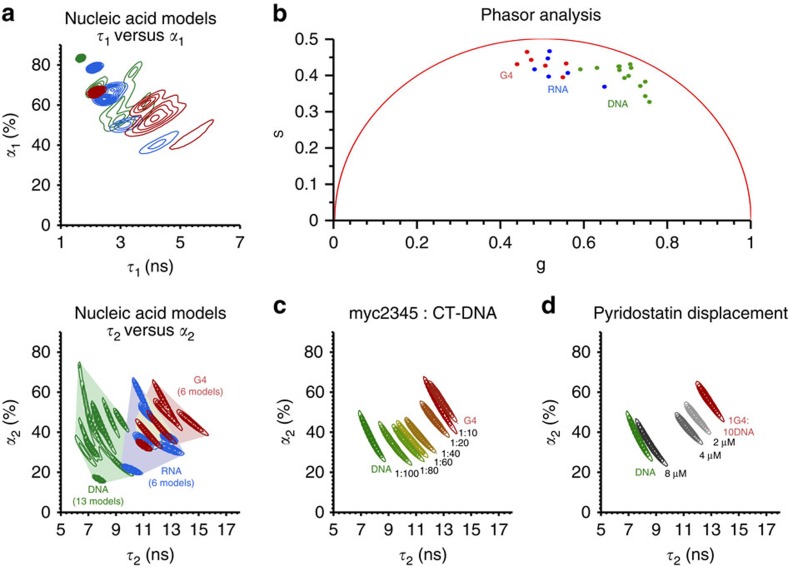
Fluorescence lifetimes of **DAOTA-M2** in the presence of different nucleic acid models in solution as determined by TCSPC-FLIM data analysis. (**a**) Contour maps of nucleic acid models for fluorescence lifetime and the corresponding amplitude for each pixel of the FLIM image (512 × 512). The difference between each contour level is 5% of the maximum bin frequency for cumulative G-quadruplexes (red), DNA (green) or RNA (blue) models. The minimum contour contains 95% of the data for each model based on the maximum bin frequency. For the *τ*_2_ versus *α*_2_ contour map boundaries are also defined for each group of nucleic acid models. (**b**) The phasor transform centroid for the FLIM image of each individual nucleic acid model, and is colour coded for G-quadruplexes (red), DNA (green) and RNA (blue) models. (**c**) The *τ*_2_ versus *α*_2_ contour maps for mixtures of G-quadruplex (myc2345) and DNA (CT-DNA). (**d**) *τ*_2_ versus *α*_2_ contour maps for displacement of **DAOTA-M2** from G-quadruplex (myc2345) with increasing concentration of G-quadruplex selective ligand pyridostatin. For all intensity decay traces, fits, residuals, *χ*_r_^2^ distributions and individual nucleic acid model contour plots, see Supplementary Fig. 8.

Interesting trends in the lifetime measurements for different nucleic acid topologies were observed. A bi-exponential decay was necessary to describe the time-resolved data for **DAOTA-M2** in the presence of the different nucleic acid structures under study. This implies that more than one ‘species' or conformation of the fluorophore exists in these samples. We note that these traces were measured in the presence of at least a twofold excess of nucleic acid compared with the end point of steady-state emission titrations. Thus, the concentration of free compound should be negligible. Furthermore, while the goodness of fit 
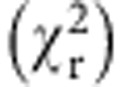
 is between 0.8 and 1.3 in all cases, it was anticipated that there would be some correlation of the lifetime and amplitude for each component to achieve the best overall fit. Therefore, for each nucleic acid topology, two-dimensional plots of *τ*_1_ versus *α*_1_ and *τ*_2_ versus *α*_2_ were created (Fig. 3a and Supplementary Fig. 8).

Of the two lifetime components, *τ*_1_/*α*_1_ offers no contrast between different nucleic acid structures (Fig. 3a). This short and similar lifetime component (*τ*_1_=1–7 ns) indicates that **DAOTA-M2** resides in an environment independent of the nucleic acid topology. Conversely, the longer *τ*_2_ offers a broad spectrum of lifetimes (6–16 ns), progressing from shorter lifetimes for dsDNA and ssDNA to significantly longer lifetimes for RNA and G-quadruplexes. Importantly, when *α*_2_ is also considered, DNA, RNA and G-quadruplex models can be clearly resolved despite partial overlap of their *τ*_2_ values.

To ensure that our observations are independent of the fitting model, all data were analysed using the phasor approach^29^. This involves plotting the real versus imaginary components of a Fourier transform of the decay for each pixel. The centroids of the resultant phasor transform clouds are shown in Fig. 3b. All points lie inside the universal circle (red line) indicating that a multi-exponential model is required to analyse the original decay data. Moreover, the separation of DNA, RNA and G-quadruplex signals is comparable to that of the *α*_2_ versus *τ*_2_ plots (Fig. 3a). This clear separation supports our previous assertion that ss/ds DNA, RNA and G-quadruplexes can be distinguished by the lifetime of bound **DAOTA-M2** and the observed correlation is fitting model independent.

In a cellular context, more dynamic mixtures of nucleic acids should be expected as opposed to regions of only DNA, RNA or G-quadruplex models. Thus, the ability of **DAOTA-M2** to detect G-quadruplexes (myc2345 was used as an example) in the presence of dsDNA (CT-DNA) was determined by titrating duplex DNA into a solution of G-quadruplex DNA containing **DAOTA-M2** (Fig. 3c). Gradual shifts to shorter *τ*_2_/*α*_2_ values were observed on increasing CT-DNA concentration as **DAOTA-M2** relocated from a G-quadruplex to duplex environment.

The ability of a non-emissive G-quadruplex ligand to displace **DAOTA-M2** from G-quadruplexes was also explored. On the addition of increasing amounts of pyridostatin (a well-established G-quadruplex binder^17^,^30^) to a mixture of G-quadruplex DNA, duplex DNA and **DAOTA-M2**, progressive shifts of *τ*_2_/*α*_2_ to lower values were observed. This is indicative of **DAOTA-M2** being displaced from G-quadruplex DNA by a more selective binder (pyridostatin), and binding to duplex DNA instead (Fig. 3d).

### Live-cell imaging with DAOTA-M2

The observed difference in fluorescence lifetimes between nucleic acid models suggested that **DAOTA-M2** could be used as an optical probe to study the interactions between a small molecule and G-quadruplexes in live cells. To explore this, cytotoxicity of **DAOTA-M2** was first established in the osteosarcoma cell line U2OS (Supplementary Fig. 9). Encouragingly, **DAOTA-M2** did not display significant cytotoxicity with an IC_50_≥100 μM over 24 h (in comparison with **TOTA** with an IC_50_=12.7±2.1 μM over 24 h). Thus, using confocal microscopy, cellular uptake was investigated using a wide range of **DAOTA-M2** incubation times (4–24 h) and non-toxic concentrations (5–20 μM) in the U2OS cell line.

**Figure 4:**
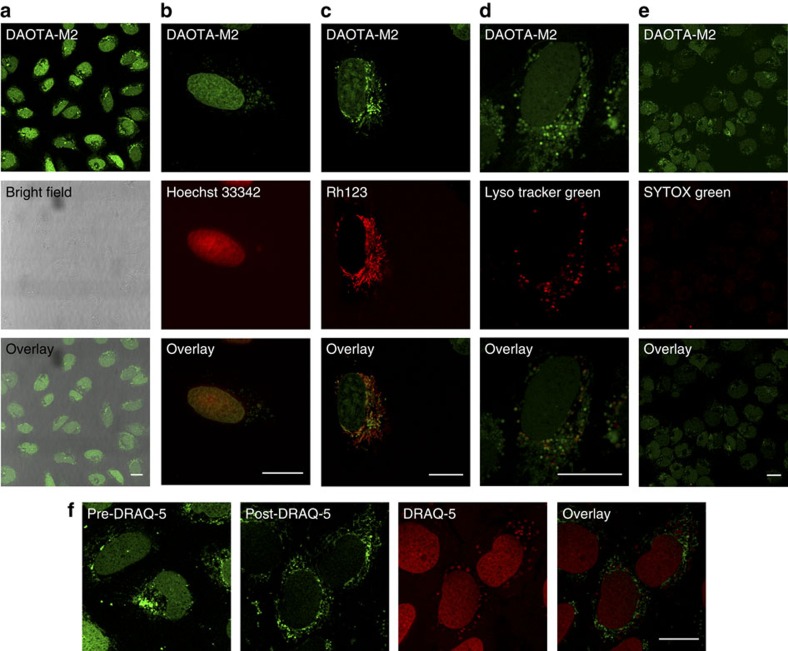
Confocal microscopy images of U2OS cells incubated with **DAOTA-M2**. (**a**) The general staining observed in combination with the transmission bright-field image (20 μM, 24 h). (**b** -**f**) Co-localization of **DAOTA-M2** (5/20 μM, 4/24 h, green) with (**b**) Hoechst 33342 (5 μM, 30 min, red), (**c**) Rh123 (5 μM, 3 min, red), (**d**) LysoTracker Green DND-26 (50 nM, 3 min, red), (**e**) SYTOX Green (2 μM, 5 min, red) and (**f**) DRAQ-5 (5 μM, 30 min, red) obtained by sequential acquisition.

Confocal microscopy showed clear nuclear staining within 4 h, which was maintained up to 24 h post treatment (Fig. 4). DNA binding was confirmed by co-localization with Hoechst 33342 (a dsDNA groove binder; Fig. 4b) and displacement of **DAOTA-M2** by DRAQ-5 (another dsDNA binder; Fig. 4f). Cytoplasmic staining showed co-regional localization with mitochondrial stain Rh123 (ref. 31), and negligible overlap with LysoTracker Green DND-26. Furthermore, it should be noted that intensity-based images displayed enhanced nuclear localization with increasing time and concentration of **DAOTA-M2** treatment. Under these experimental conditions, no photobleaching was observed and cell viability was not compromised; SYTOX Green, a cell impermeable dye, did not generate strong nuclear staining (Fig. 4e).

**Figure 5:**
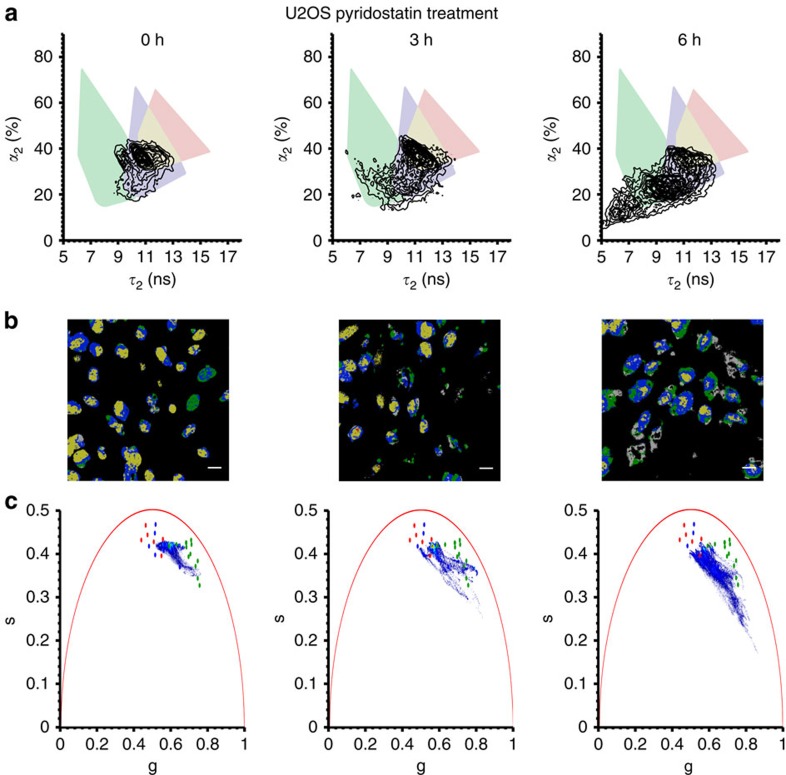
FLIM visualization of U2OS cells incubated with **DAOTA-M2** (20 μM, 24 h) and subsequently treated with G-quadruplex selective ligand pyridostatin (10 μM) over time (0–6 h). (**a**) Cellular *τ*_2_ and *α*_2_ plotted in the form of a contour map and superimposed on *in vitro* nucleic acid models boundaries for G-quadruplexes (red), DNA (green), RNA (blue) or overlapping RNA/G-quadruplexes (yellow). The difference between each contour level is 5% of the maximum bin frequency for the cellular data and the minimum contour contains 95% of the data relative to the maximum bin frequency. (**b**) Images are colour coded based on the location of the cellular pixel *τ*_2_ and *α*_2_ with respect to the *in vitro* models boundaries. Scale bars, 20 μm. (**c**) Phasor-transformed cellular fluorescence decays as a heat map of pixels (red—highest frequency, blue—lowest frequency) superimposed on *in vitro* colour-coded nucleic acid models. See Supplementary Fig. 10 for *χ*_r_^2^, representative decay traces and fits for all data. For data analysis excluding cytoplasmic staining, see Supplementary Fig. 11.

To explore whether the compound binds to G-quadruplexes in live cells, FLIM was carried out. Fluorescence decay traces were collected in every pixel of the cellular images captured to study potential differences in the probe's lifetime, which in turn provide a measure of the nucleic acid topology to which **DAOTA-M2** was bound (Fig. 5).

As with the *in vitro* studies (Fig. 3), individual decays in FLIM images of live cells could be accurately fitted by a bi-exponential function. Since *τ*_2_ versus *α*_2_ showed the clearest ability to distinguish *in vitro* between G-quadruplexes and other nucleic acid topologies, 95% minimum contour boundaries were defined for each group—DNA, RNA and G-quadruplex—and were colour coded green, blue and red, respectively, while overlapping RNA/G-quadruplex points are shown in yellow. Cellular *α*_2_ versus *τ*_2_ data was superimposed on this colour-coded contour plot (Fig. 5a). Cellular pixels within the defined boundaries were then assigned the appropriate colour to generate colour images (Fig. 5b). In general, the range of *τ*_2_/*α*_2_ from cells falls within the confines of the model systems, and are at the interface between the RNA and G-quadruplex topologies.

Next, U2OS cells were treated with the non-fluorescent G-quadruplex selective ligand pyridostatin. Based on *in vitro* models (Fig. 3d), shifts to shorter *τ*_2_/*α*_2_ values would be expected if pyridostatin displaces **DAOTA-M2** from G-quadruplexes. Encouragingly, with increasing pyridostatin incubation times, lower *τ*_2_/*α*_2_ values were observed (Fig. 5) in addition to a decrease in total fluorescence intensity. Furthermore, treatment of cells with non-specific nucleic acid damaging agent cisplatin for 24 h failed to generate any significant change in the *τ*_2_/*α*_2_ values of **DAOTA-M2** (Supplementary Figs 12 and 13). Even though the concentration of G-quadruplexes in live cells is expected to be low, our method suggests a change in relative abundance of **DAOTA-M2**—quadruplex interactions on addition of the competitor ligand pyridostatin. However, it should be noted that owing to the reduction in fluorescence intensity, the sensitivity of this method was impaired and longer acquisition times were necessary. Consequently, challenges still remain to apply **DAOTA-M2** as a general probe for G-quadruplex displacement assays in live cells.

## Discussion

There is increasing experimental evidence suggesting that G-quadruplexes play essential roles in key biological processes. As a result, there has been a growing demand for tools to study these non-canonical DNA structures *in vivo*. While antibodies have been successfully used to image G-quadruplexes in fixed cells, live-cell studies are of vital importance in rigorously addressing the dynamic cause and effect of these structures *in vivo*. Therefore, the development of cell-permeable small-molecule optical probes for use in real-time live-cell imaging is highly desirable. In this paper, we have shown that the triangulenium core provides a versatile platform for the design of fluorescent probes. Through the incorporation of essential design features of a good G-quadruplex binder, a novel DNA ‘turn-on' probe **DAOTA-M2** has been developed. A key feature of this probe is that its emissive lifetime changes considerably upon interaction with different nucleic acid topologies. In particular, significantly higher *τ*_2_/*α*_2_ values were observed upon interaction of **DAOTA-M2** with a wide range of G-quadruplexes, compared with dsDNA or ssDNA. We note that there is some overlap of the probe's *τ*_2_/*α*_2_ values upon its interaction with G-quadruplexes and RNA—although the values for G-quadruplexes are still consistently higher. Although this is not ideal—and will indeed need to be optimized in the future—the differences are large enough to be detectable.

While a two-component model accurately describes all our data, the origin of *τ*_1_ and *τ*_2_ is not clear for each nucleic acid structure. There are two equally plausible explanations for these observations. The first is that the compound resides in two distinct binding pockets with distinct local environments (for example, groove binding and intercalation/*π*–*π* stacking). The second is that the compound itself has different excited state lifetimes, and resides in one or more highly similar binding pockets that can be adequately described by *τ*_1_ and *τ*_2_ (for example, two G-tetrads in the same G-quadruplex, intercalation between different base pairs in duplex DNA). The latter interpretation correlates well with the intramolecular PET transfer quenching observed in purely aqueous systems.

The favourable nuclear localization and minimal toxicity of this new compound also enabled us to carry out FLIM studies in live cells. Our FLIM data suggest that **DAOTA-M2** does interact with G-quadruplexes in live cells, as evidenced by high *τ*_2_/*α*_2_ values observed for this dye in the nuclei of live cells. This is supported by preliminary data that demonstrate a real-time decrease in **DAOTA-M2** *τ*_2_/*α*_2_ values upon addition of pyridostatin—a stronger G-quadruplex binder. To confirm that these observations are due to G-quadruplex interactions as opposed to DNA damage subsequently induced by pyridostatin, a control experiment was performed using cisplatin. Even after 24 h incubation with this non-specific nucleic acid damaging agent, no significant change in the *τ*_2_/*α*_2_ values of **DAOTA-M2** was observed. This indicates that DNA damage is not responsible for the *τ*_2_/*α*_2_ changes observed upon pyridostatin treatment, and suggests that our methodology could potentially be used to probe the dynamics of small-molecule/G-quadruplexes interactions in live cells.

This work builds on the growing evidence that small molecules can indeed interact with G-quadruplexes *in cellulo*, and shows that a useful degree of binding is achievable based on current G-quadruplex ligand design paradigms. Our future efforts will concentrate on designing fluorescence lifetime-based probes that are capable of achieving greater signal differentiation between G-quadruplex- and RNA-binding sites.

## Methods

Full synthetic procedures and characterization of new compounds can be found in Supplementary Methods.

### General procedures

Absorption measurements were made on a Perkin-Elmer ultraviolet-visible spectrometer. Emission spectra were obtained on a Varian Cary-Eclipse or Horiba Jobin Yvon Fluorolog fluorescence spectrometer. The oligonucleotides used were purchased RP-cartridge purified from Eurogentec. CT-DNA was obtained from Sigma-Aldrich. pH was measured using a Mettler Toledo pH meter.

### Quantum yield

At least six absorption and emission spectra were recorded of the compound at different concentrations in aerated 10 mM lithium cacodylate buffer (pH 7.3) containing 100 mM potassium chloride. The absorbance was below 0.1 at wavelengths above the excitation wavelength (436 nm) and emission was collected from 500 to 800 nm. [Ru(byp)_3_Cl_2_] in aerated Milli-Q water was used as the reference standard (Φ_Ru_=0.028; ref. 32). A plot of absorbance (436 nm) versus integrated emission intensity yielded a linear fit to the equation *y*=*mx*+*c* (*R*^2^>0.99). The quantum yield of the compound was determined by Φ_*x*_=Φ_Ru_(*m*_*x*_/*m*_Ru_).

### Solvent effects emission spectra

**TOTA**, **ADOTA-M** and **DAOTA-M2** (2 μM) were excited at 404 nm where absorbance is minimal and emission was monitored from 500 to 750 nm. Fluorescence enhancement or quenching was determined by the integrated fluorescence of the emission spectrum. pH 1.0 and 7.3 measurements were recorded in 0.1 M HCl and 10 mM lithium cacodylate buffer containing 100 mM KCl, respectively. Measurements in 1,4-dioxane were recorded at pH 7.3 (10 mM lithium cacodylate buffer containing 100 mM KCl). Ionic strength measurements were performed in 10 mM lithium cacodylate buffer (pH 7.3) by increasing KCl concentration from 0 to 220 mM.

### Time-correlated single photon counting

Time-resolved fluorescence decay traces were obtained using a TCSPC Jobin Yvon IBH data station (5000F, HORIBA Scientific Ltd) using a 404-nm excitation source (full width of half-maximum intensity (FWHM)=200 ps). Samples were prepared as detailed under Solvent effects emission spectra in Methods section. Decays were recorded at the emission wavelengths 520, 555 or 575 nm (±16 nm) for **TOTA**, **ADOTA-M** and **DAOTA-M2**, respectively, using emission monochromator. Signal intensity was at least 10,000 counts in the peak maximum. Two long-pass filters (>490, 550 or 570 nm long pass) were used in the detection channel to avoid light scattering for fluorescence decays. A neutral density filter was used for the instrument response function (IRF) measurements using a Ludox solution, detecting emission at the excitation wavelength. Traces were fitted by iterative reconvolution to the equation 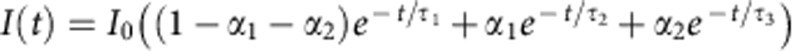 where *α*_1_ and *α*_2_ are variables and 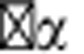 is normalized to unity. The fractional contribution to the steady-state emission is calculated from the equation 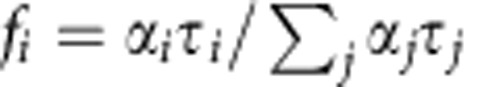. The average lifetime was calculated using the equation 
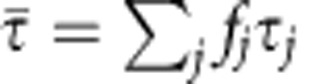
. For mono- or bi-exponential decays, the *α*_1_ and/or *α*_1_ terms were set to zero. In the case of solvent polarity, to gain greater accuracy in the lifetimes obtained, a tri-exponential decay model was fitted to decay traces with different 1,4-dioxane percentages using a common *τ*_1_, *τ*_2_ and *τ*_3_ (global analysis)^33^. A prompt shift was included in the fitting to take into account differences in the emission wavelength between the IRF and decay as well as the different number of filters used. The goodness of fit was judged by consideration of the deviations from the model via a weighted residuals plot. Least square minimization was performed using the Quasi-Newton algorithm in MatLab (R2013a).

### Excited state p*K*a* determination

**ADOTA-M** and **DAOTA-M2** (2 μM, 10 mM sodium phosphate buffer and 0.1 M KCl (pH 7.6)) were titrated with citric acid (3 M) from pH 7.6 to 3 (phosphate–citric acid buffer capacity=2.6–7.6; ref. 34). The pH was read before the emission spectrum was recorded (excitation wavelength=404 nm (minimal absorbance), emission wavelength=500–750 nm). The pH was then plotted against the numerical derivative *δ*F/*δ*pH, where F is the integrated emission. The minimum was then determined to identify p*K*a*. Three independent repeats were performed.

### List of nucleic acid models

The oligonucleotides used in this study and their corresponding molar extinction coefficients are summarized in Table 2.

### DNA annealing

Oligonucleotides were dissolved in buffer containing 100 mM potassium chloride and annealed at 95 °C for 5 min before cooling to room temperature overnight on a heat block. Annealing concentrations were ∼50–100 μM (except for TBA and myc2345, which were ∼1 mM). For PDGF-A, as reported previously, the oligonucleotide was annealed in the same buffer but containing only 25 mM potassium chloride at 15 μM concentrations to enable intramolecular folding^35^. For d(GC)_8_, samples were annealed in buffer containing 5 M sodium chloride to form Z-DNA stock samples^36^. For Z-DNA measurements, this salt concentration was maintained. For normal B-form measurements of d(GC)_8_, the sodium chloride concentration was reduced to 250 mM. CT-DNA and transfer RNA (tRNA) was dissolved in buffer containing 100 mM potassium chloride and their concentration was checked using the molar extinction coefficients 13,200 (base pair) and 8,250 (base) mol dm^−3^ cm^−1^, respectively. Double- and single-stranded nucleic acid model concentrations were converted to base and base pair concentration by multiplying the number of base/base pairs in the sequence.

### Absorption and emission titrations

Compound concentration was held constant (20 and 2 μM for absorption and emission measurements, respectively) in 10 mM lithium cacodylate buffer (pH 7.3) containing 100 mM potassium chloride. Concentrated DNA was then titrated to the compound solution until changes in the absorbance/emission became noticeably indifferent. Fold enhancement of emission was determined by the fluorescence at the end of the titration divided by the initial fluorescence of the compound-only spectrum. Samples were excited at 465 nm, where upon titration of DNA, absorbance changes were negligible (<0.005) as determined by ultraviolet-visible spectroscopic titrations. Emission spectra were recorded from 500 to 800 nm. Three independent repeats were performed. PDGF-A absorption titrations could not be performed due to the low concentration annealing conditions required for intramolecular G-quadruplex formation. Emission titrations were performed by the addition of PDGF-A (15 μM) containing **DAOTA-M2** (2 μM) to a compound-only solution. **DAOTA-M2** was added to the PDGF-A DNA to correct for dilution of **DAOTA-M2** upon DNA titration. Titration curves were fitted using 1:1 binding model using the absorbance or emission values at 554–504 nm and 584–634 nm using a modified form of the MatLab (R2013a) script reported previously^37^. The trust-region-reflective minimization algorithm was used. Binding constants are only reported for emission titrations since the ratio of the initial compound concentration to the calculated absorbance dissociation constants was ∼100 and therefore more error-prone. Instead, the sudden change in gradient of the absorption binding curves was used to determine the stoichiometry of binding (mole ratio method)^38^. This was a ratio of two compounds per five base pairs (CT-DNA and ds17), one to five bases for ss17, one-to-one G-quadruplex (TBA) and two compounds to one G-quadruplex (myc2345 and PDGF-A). Association constants were then determined relative to the DNA binding motif as defined by the stoichiometry. For example, in the case of ss17, the binding constant is reported relative to a binding of one compound to a motif of five bases as opposed to one base. This is based on the assumption that there is no co-operativity in binding and enables all data to be modelled by a one-to-one compound to DNA binding motif stoichiometry^39^.

### Fluorescence lifetime nucleic acid models

The oligonucleotides used in this study are detailed under List of Nucleic Acid Models in Methods section . The concentration of **DAOTA-M2** relative to nucleic acid was chosen based on DNA emission titrations to ensure minimal unbound compound (**DAOTA-M2=**2 μM, double-stranded models=40 μM (bp), single-stranded models=60 μM (base), G-quadruplex models=4 μM (strand)). The exceptions were tRNA, GCss17a RNA and d(A)_17_ where a larger excess of nucleic acid was used (120 μM, base) as well as dsGA Mismatch (80 μM, base pair). The buffer system was 10 mM lithium cacodylate buffer (pH 7.3) containing 100 mM potassium chloride with the exception of Z-DNA and d(GC)_8_ for which salt concentration was 5 M and 250 mM sodium chloride, respectively.

### Cell culture

Bone osteosarcoma U2OS cells (obtained from London Research Institute, Cancer Research UK) were grown in low glucose phenol red-free Dulbecco's modified Eagle medium containing 10% fetal bovine serum at 37 °C with 10% CO_2_ in humidified air. Cells were seeded on chambered coverglass (ca. 2 × 10^4^, 200 μl, 0.8 cm^2^) for 6–24 h before the media was replaced with fresh media containing **DAOTA-M2** for the specified period of time and concentration (5–20 μM, 4–24 h, 200 μl). Before imaging, the cells were washed with PBS and the incubation medium was replaced with fresh growth media. For pyridostatin treatment, cells were incubated with **DAOTA-M2** for 24 h before being washed with PBS and grown in media containing pyridostatin (10 μM, 200 μl) for the specified times. For cisplatin treatment, cells were co-incubated with **DAOTA-M2** (20 μM) and cisplatin (12.5 and 25 μM, 200 μl) for 24 h in medium containing HEPES (25 mM). Cells were then washed with PBS and fresh media were added before imaging. For co-localization experiments, cells were washed with PBS and then incubated with Rh123 (5 μM, 3 min), Hoechst 33342 (5 μM, 30 min), DRAQ-5 (5 μM, 5 min) or LysoTracker Green DND-26 (50 nM, 3 min) in PBS (200 μl). Cells were then washed with PBS and fresh media were added before imaging. In the case of SYTOX Green (2 μM, 5 min, 200 μl PBS), cells were not washed after the addition of the dye.

### Cytoxicity assay

Cells were seeded at a density of 5 × 10^3^ per well of a 96-well plate (50 μl). After 24 h, compounds were added at the appropriate concentration in triplicate (total volume 100 μl). After a further 24 h, 20 μl of the MTS Assay reagent was added according to the Promega MTS Assay protocol^40^. Absorbance at 490 nm (cisplatin, **ADOTA-M** and **DAOTA-M2**) or 510 nm (**TOTA**) was recorded 2–4 h after the addition of the reagent. In the case of **ADOTA-M** and **DAOTA-M2**, cell treatments were corrected for compound absorbance by subtracting the compound-only control run in parallel. Absolute IC_50_ was determined from the equation IC_50_=IC_100_+IC_0_/2, where IC_0_ is the absorbance of only media and IC_100_ is the absorbance of DMSO-only-treated cells. Three independent repeats were performed.

### Confocal microscopy

Cell treatment conditions can be found under Cell culture in Methods section. Confocal imaging of **DAOTA-M2** was performed using a confocal laser-scanning microscope (Leica TCS SP5) with a × 63 (numerical aperture (NA) 1.2) HCX PL APO CS water immersion objective lens with correction collar (11506279, Leica Microsystems, Ltd). The samples were excited using internal microscope lasers and emission intensity was recorded at the appropriate emission wavelength. For co-localization experiments, fluorescence images were sequentially acquired. For DRAQ-5 co-localization, **DAOTA-M2** was excited at 458 nm and its emission was recorded from 550 to 600 nm, whereas DRAQ-5 was excited at 633 nm and its emission was recorded from 750 to 800 nm. For Hoechst 33342 co-localization, **DAOTA-M2** was excited at 514 nm and its emission was recorded from 600 to 700 nm, whereas Hoechst 33342 was excited at 405 nm and its emission was recorded from 450 to 500 nm. For Rh123, SYTOX Green and Lysotracker Green DND-26 co-localization, **DAOTA-M2** was excited at 561 nm and its emission was recorded from 700 to 759 nm, whereas the dyes were excited at 488 nm and their emission was recorded from 510 to 530 nm.

### Fluorescence lifetime imaging microscopy

Cell treatment conditions can be found under Cell Culture in Methods section. FLIM imaging of **DAOTA-M2** was performed using a pulsed diode laser (Hamamatsu, excitation wavelength=467 nm, repetition rate=10 MHz) coupled into an inverted confocal microscope (Leica TCS SP2) with a × 63(NA 1.2) water or × 63 (NA 1.4) oil objective. The emission (500–750 nm) was collected using a cooled Becker&Hickl HPM100-40 hybrid detector, and TCSPC was performed by a SPC-150 Becker&Hickl module. Images of 512 × 512 pixel resolution were captured. The IRF was recorded at 467 nm using fluorescein quenched by a saturated aqueous solution of sodium iodide^41^. DNA models and cellular images were acquired using the same settings, and the acquisition times was ca. 300 s (models) and 900 s (live cells). Lifetime data were fitted to a bi-exponential function for each pixel using Tri2 software^42^ ( https://www.assembla.com/spaces/ATD_TRI/wiki). To improve the signal-to-noise ratio, a 21 × 21 or 7 × 7 pixel smoothing (bin=10 or 3) was used to increase signal intensity for nucleic acid models and cellular images, respectively. For cellular images, a lower bin size and longer acquisition time were used to retain greater spatial resolution of fluorescence decays. Appropriate bin sizes were chosen to ensure a peak count of at least 750 for accurate fitting of bi-exponential traces. Data were then exported and the parameters for each pixel correlated using MatLab (R2013a). Two-dimensional contour maps were constructed with 0.1 ns or 1% amplitude bins. The total volume of the contour plot was normalized to one for each model per plot. For example in Fig. 3a, the volume under the combined G-quadruplex models was set to one and this was repeated for the combined RNA and combined DNA models. Thus, the spread of data is directly comparable. The difference between each contour level is 5% of the maximum bin frequency for the data set and the minimum contour contains 95% of the data relative to the maximum bin frequency. For cellular images, colours were assigned to each pixel based on both the amplitude and lifetime with respect to the *in vitro* model data for ssDNA/dsDNA (green), RNA (blue) and G-quadruplex (red) models (as well as overlapping G-quadruplex/RNA models, yellow) to generate a 512 × 512 image.

### Phasor analysis

Phasor analysis was performed on FLIM images using software written in-house in MatLab (MathWorks). Phasor analysis is a Fourier domain technique that involves plotting the real and imaginary components of the Fourier transform of a fluorescence decay^29^ to provide an alternative two-dimensional representation of the data. The real (*g*) and imaginary (*s*) components are calculated as:


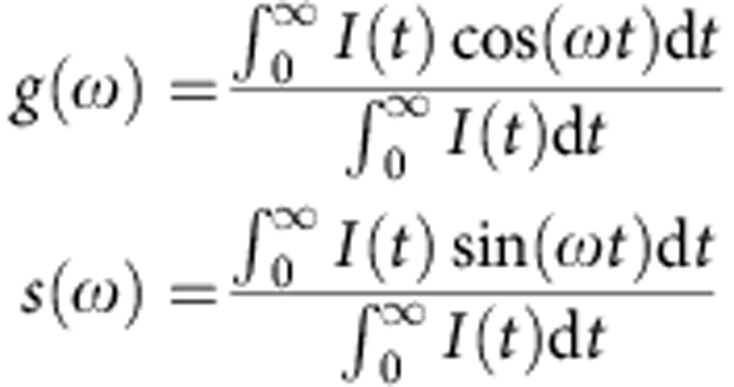


Here *t* is time, *I*(*t*) is the measured fluorescence decay profile and *ω* is a parameter that was set to 2*π* × 16.6 MHz to have the optimal spread of phasor points obtained from decays with different lifetimes. If the data are measured with a frequency domain FLIM technique, *ω* has to be equal to the angular frequency of excitation laser. Phasors of mono-exponential decays lie on the ‘universal circle' (a semicircle centred at *g*=0.5, *s*=0), whereas multi-exponential decays produce phasors inside the universal circle. For each fluorescence lifetime image, the phasor transform appears as a diffuse cloud indicating the spread of fluorescence lifetimes. To simplify the visualization of results, where appropriate, the weighted centroid of the phasor cloud was calculated using the MatLab ‘regionprops'.

## Additional information

**How to cite this article:** Shivalingam, A. *et al.* The interactions between a small molecule and G-quadruplexes are visualized by fluorescence lifetime imaging microscopy. *Nat. Commun.* 6:8178 doi: 10.1038/ncomms9178 (2015).

## Supplementary Material

Supplementary Information Supplementary Figures 1-13, Supplementary Methods and Supplementary References

## Figures and Tables

**Table 1:** Photophysical properties of **TOTA**, **ADOTA-M** and **DAOTA-M2** in different local aqueous environments.

	***λ***_**em,max**_ **(nm)**	***F*****/*****F***_**pH7.3**_	***τ***_**1**_ **(ns/f)**	***τ***_**2**_ **(ns/f)**	***τ***_**3**_ **(ns/f)**
TOTA*					
pH 1.0	519	0.7	8.5/1.00	—	—
pH 7.3	519	1	8.6/1.00	—	—
					
**ADOTA-M**					
pH 1.0	550	10.6	20.5/1.00	—	—
pH 7.3	555	1	0.4/0.20	18.3/0.80	—
50% 1,4-dioxane	560	0.7	0.1/0.11	3.1/0.05	20.1/0.84
					
**DAOTA-M2**					
pH 1.0	572	13.7	18.3/1.00	—	—
pH 7.3	581	1	1.3/0.92	3.7/0.08	—
50% 1,4-dioxane	590	0.7	0.5/0.53	1.1/0.38	5.7/0.08

Measurements in 50% 1,4-dioxane were recorded at pH 7.3. pH 1.0 and 7.3 measurements were recorded in 0.1 M HCl and 10 mM lithium cacodylate buffer containing 100 mM KCl (pH 7.3).

^*^The effect of solvent polarity on **TOTA** was not explored due to the dynamic quenching observed in the presence of chloride ions. See Supplementary Figs 1–4 for compound absorption/emission spectra, **TOTA** Stern–Volmer quenching plots and TCSPC decay fit residuals respectively.

**Table 2:** Sequences of oligonucleotides used in this study with their corresponding molar extinction coefficients.

**Code**	**Sequence (5′–3′)**	**Molar extinction co-efficient (mol dm**^−3^ **cm**^−1^)
*Quadruplexes*		
HT-G4	TTGGGTTAGGGTTAGGGTTAGGGA	244,300
TBA	GGTTGGTGTGGTTGG	143,300
myc2345	TGAGGGTGGGGAGGGTGGGGAA	229,900
PDGF-A	GGAGGCGGGGGGGGGGGGGCGGGG GCGGGGGCGGGGGAGGGGCGCGGC	467,400
BCL2 (RNA)	AGGGGGCCGUGGGGUGGGAGCUGGGG	257,600
ckit87up	AGGGAGGGCGCTGGGAGGAGGG	226,700
		
*dsDNA/ssDNA*		
ds17	Equimolar CCAGTTCGTAGTAACCC and GGGTTACTACGAACTGG	160,900 and 167,400
ATds17	Equimolar CTATTGCATACTAATTC and GAATTAGTATGCAATAG	161,000 and 181,500
ss17	CCAGTTCGTAGTAACCC	160,900
ATss17a	CTATTGCATACTAATTC	161,000
dsGA Match	Equimolar TCGTTCACTC and GAGTGAACGA	85,800 and 109,400
dsGA Mismatch	GAGTGAACGA	109,400
dsmyc	Equimolar TTCCCCACCCTCCCCACCCTCA and TGAGGGTGGGGAGGGTGGGGAA	179,900 and 229,900
d(T)_17_·d(A)_17_	Equimolar TTTTTTTTTTTTTTTTT and AAAAAAAAAAAAAAAAA	138,300 and 207,400
d(A)_17_	AAAAAAAAAAAAAAAAA	207,400
d(AT)_8_	ATATATATATATATAT	177,500
d(GC)_8_	GCGCGCGCGCGCGCGC	134,500
Z-DNA	GCGCGCGCGCGCGCGC	134,500
CT-DNA	—	13,200
		
*dsRNA/ssRNA*		
GCds17 RNA	Equimolar GGGUUACUACGAACUGG and CCAGUUCGUAGUAACCC	164,900 and 155,700
GCds17 Hybrid	Equimolar CCAGUUCGUAGUAACCC and GGGTTACTACGAACTGG	155,700 and 167,400
ATds17 Hybrid	Equimolar CUAUUGCAUACUAAUUC and GAATTAGTATGCAATAG	169,200 and 181,500
ATss17a RNA	CUAUUGCAUACUAAUUC	169,200
GCss17a RNA	CCAGUUCGUAGUAACCC	164,900
tRNA	—	8,250
