# Supplementary material for: The interactions between a small molecule and G-quadruplexes are visualized by fluorescence lifetime imaging microscopy
Source: Nat Commun. 2015 Sep 9;6:8178. doi: 10.1038/ncomms9178 (PMC4579598; doi:10.1038/ncomms9178)
Supplement: Supplementary Information — Supplementary Figures 1-13, Supplementary Methods and Supplementary References [file ncomms9178-s1.pdf]

Supplementary figures

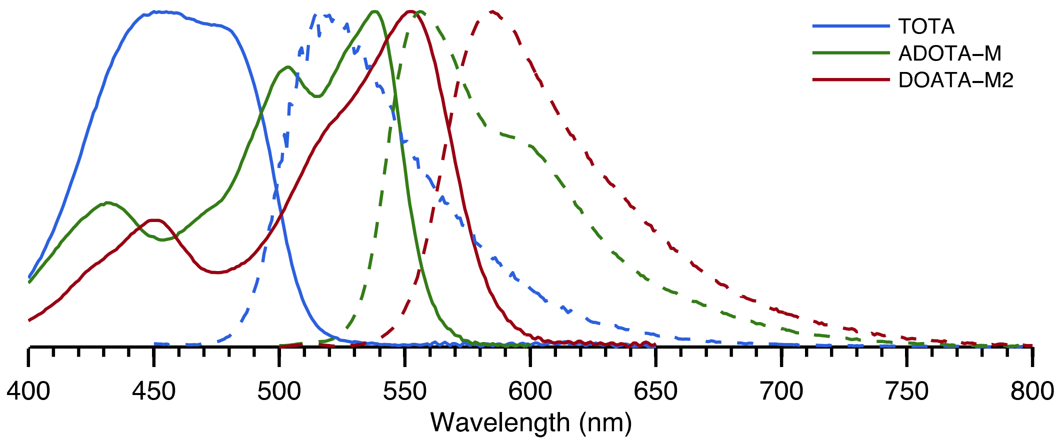

Supplementary Figure 1. Normalised absorption (solid lines) and emission (dashed lines) spectra recorded in 10 mM lithium cacodylate buffer containing 100 mM KCl (pH 7.3).

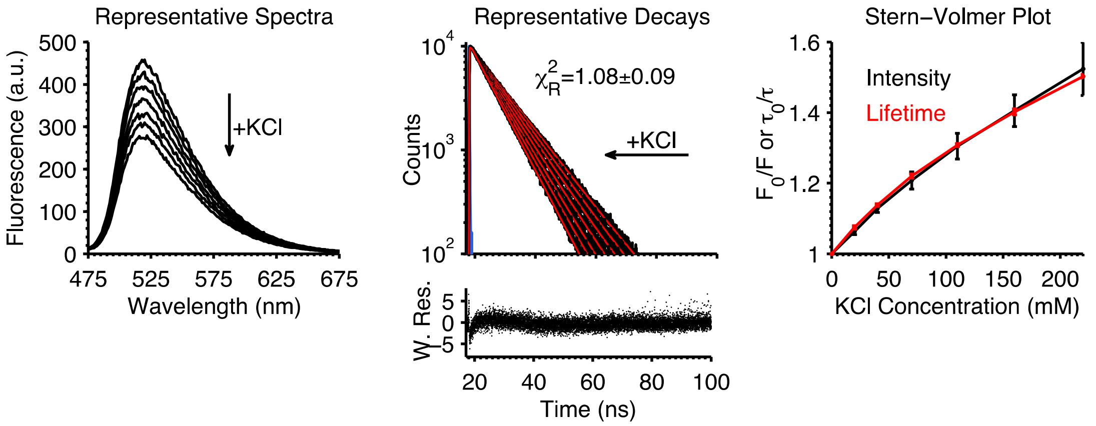

Supplementary Figure 2. Dynamic quenching of **TOTA** (2 µM, 10 mM lithium cacodylate buffer, pH 7.3) fluorescence upon increasing ionic strength (KCl). For representative decays, each decay trace is fitted to a mono-exponential model and the average χ_R^2 and standard deviation for all seven decays traces is shown. Data traces, the instrument response and fittings are shown in black, blue and red respectively. For the Stern-Volmer plot, integrated fluorescence (475-750 nm) is used and error bars represent the standard deviation of three independent repeats.

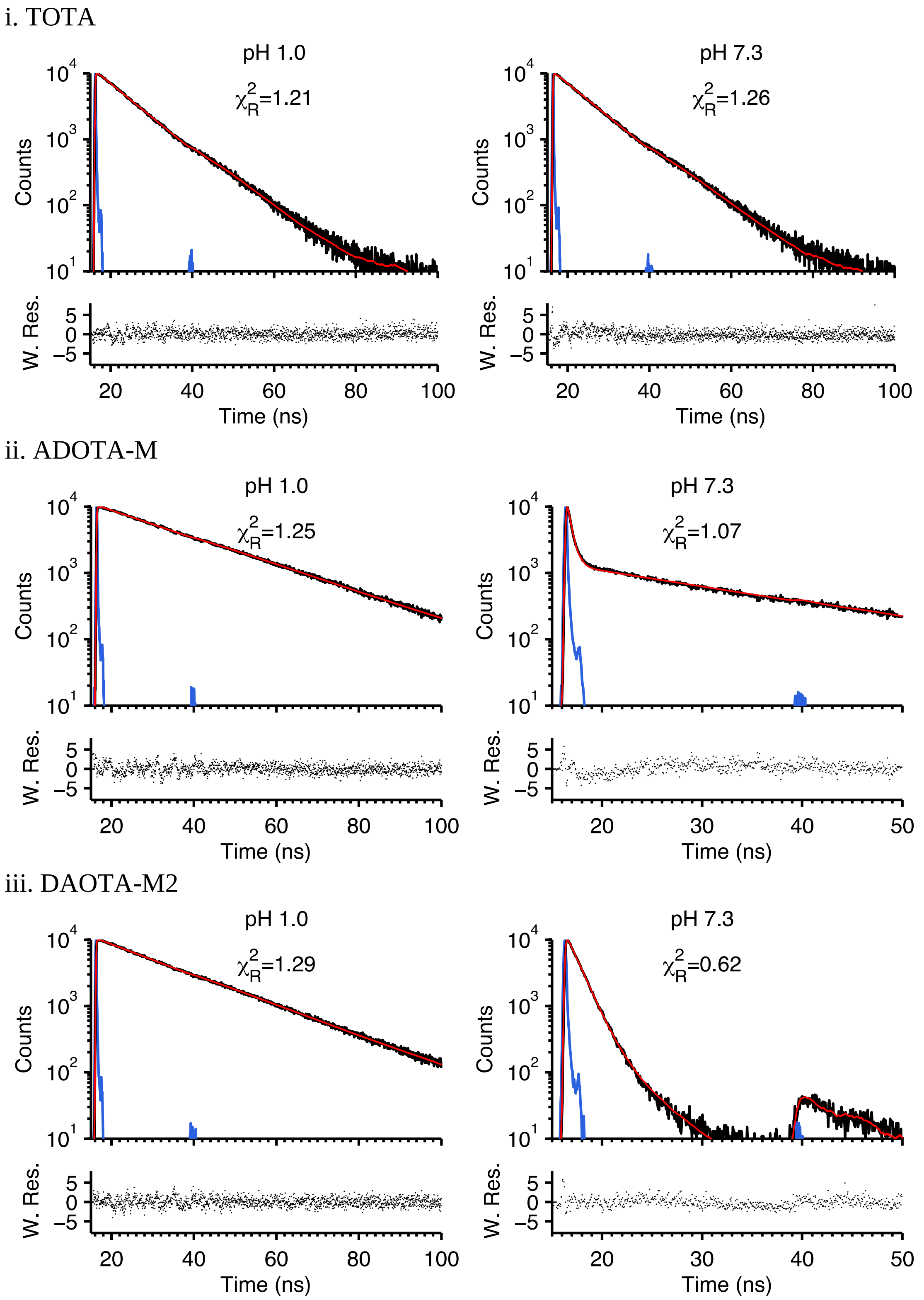

Supplementary Figure 3. The effect of pH on the fluorescence lifetime of **TOTA**, **ADOTA-M** and **DAOTA-M2**. pH 1.0 and 7.3 measurements were recorded in 0.1 M HCl and 10 mM lithium cacodylate buffer containing 100 mM KCl (pH 7.3). All fittings are to a mono-exponential decay model except for **ADOTA-M** and **DAOTA-M2** at pH 7.3 which is fitted to a bi-exponential decay model. The second component for **DAOTA-M2** very small but is evident in the residuals when fitted to a mono-exponential decay. Data traces, the instrument response and fittings are shown in

black, blue and red respectively. For numerical lifetime / fractional contribution values see main text Table 1.

i. ADOTA-M

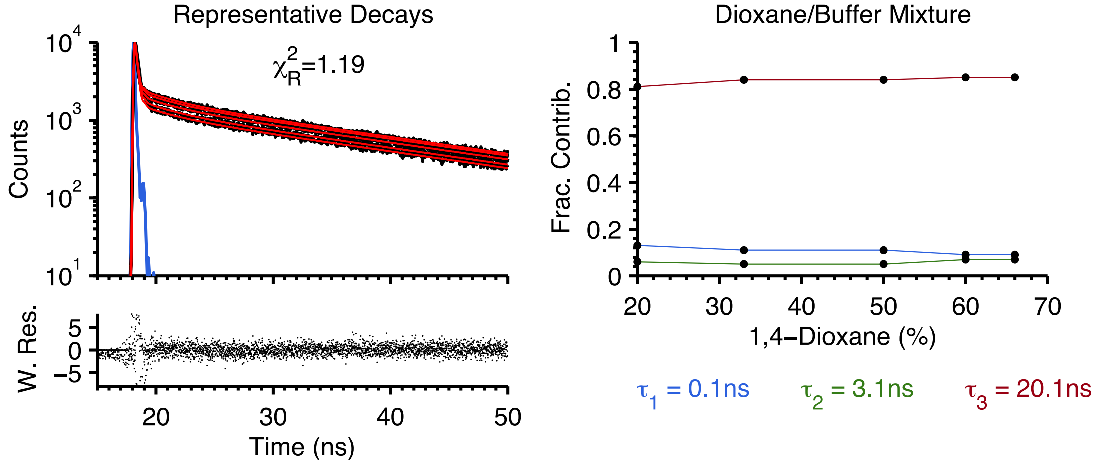

ii. DAOTA-M2

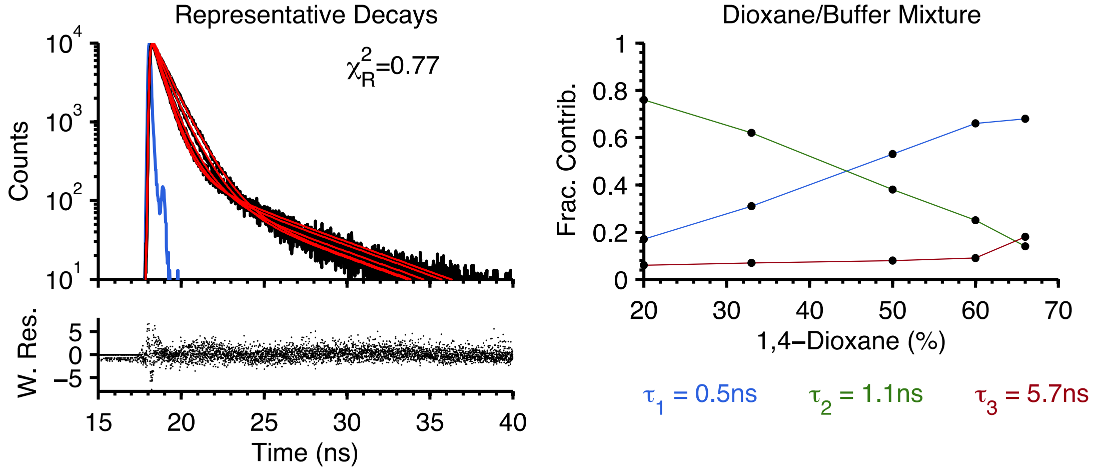

Supplementary Figure 4. The effect of solvent polarity on the fluorescence lifetime of **ADOTA-M** and **DAOTA-M2** using global fitting for $\tau_1, \tau_2$and τ_3. Measurements were recorded in 10 mM lithium cacodylate buffer containing 100 mM KCl with varying v/v 1,4-dioxane. For the representative decays plot, data traces, the instrument response and fittings are shown in black, blue and red respectively. For the fractional contribution plot, $\tau_1, \tau_2$and τ_3 are color coded with fractional contributions. Note that the lines are not fittings and are only present for clarity.

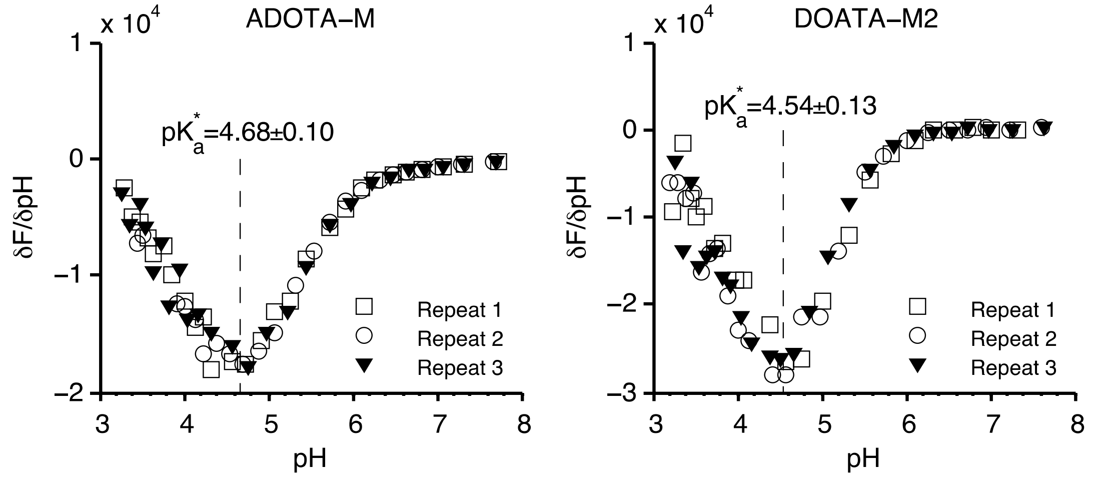

Supplementary Figure 5. Excited state pK_a* determination from the pH dependent steady state emission of **ADOTA-M** and **DAOTA-M2**. The minimum of $\delta F/\delta$pH represents the pK_a* in a 10 mM sodium phosphate – citric acid buffer system containing 100mM KCl. Errors represent the standard deviation of three repeats.

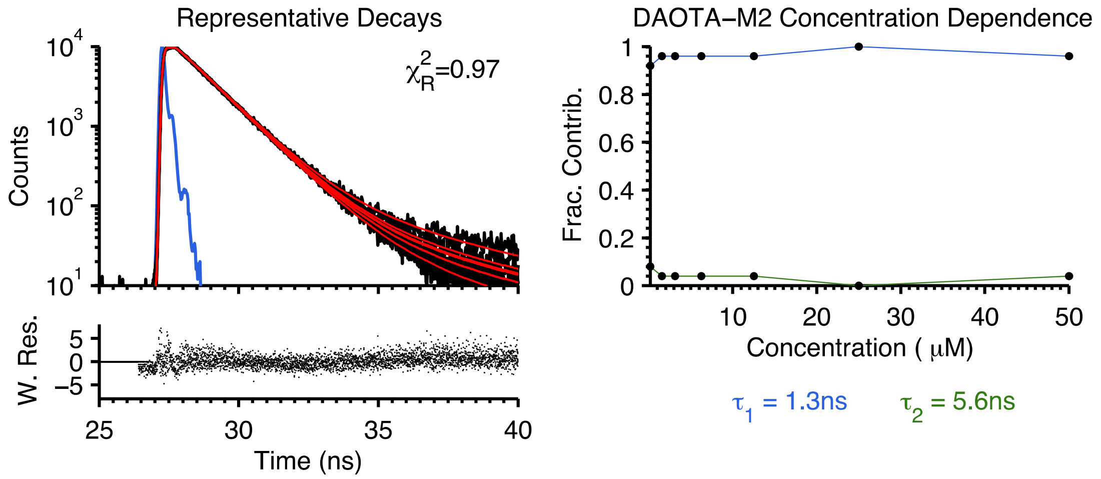

Supplementary Figure 6. The effect of **DATOA-M2** concentration (0.2 – 50 μM) on its fluorescence using global fitting for τ_1 and τ_2. Measurements were recorded in 10 mM lithium cacodylate buffer containing 100 mM KCl. For the representative decays plot, data traces, the instrument response and fittings are shown in black, blue and red respectively. For the fractional contribution plot, τ_1 and τ_2 are color coded with fractional contributions. Note that the lines are not fits and are only present for clarity.

i. CT-DNA

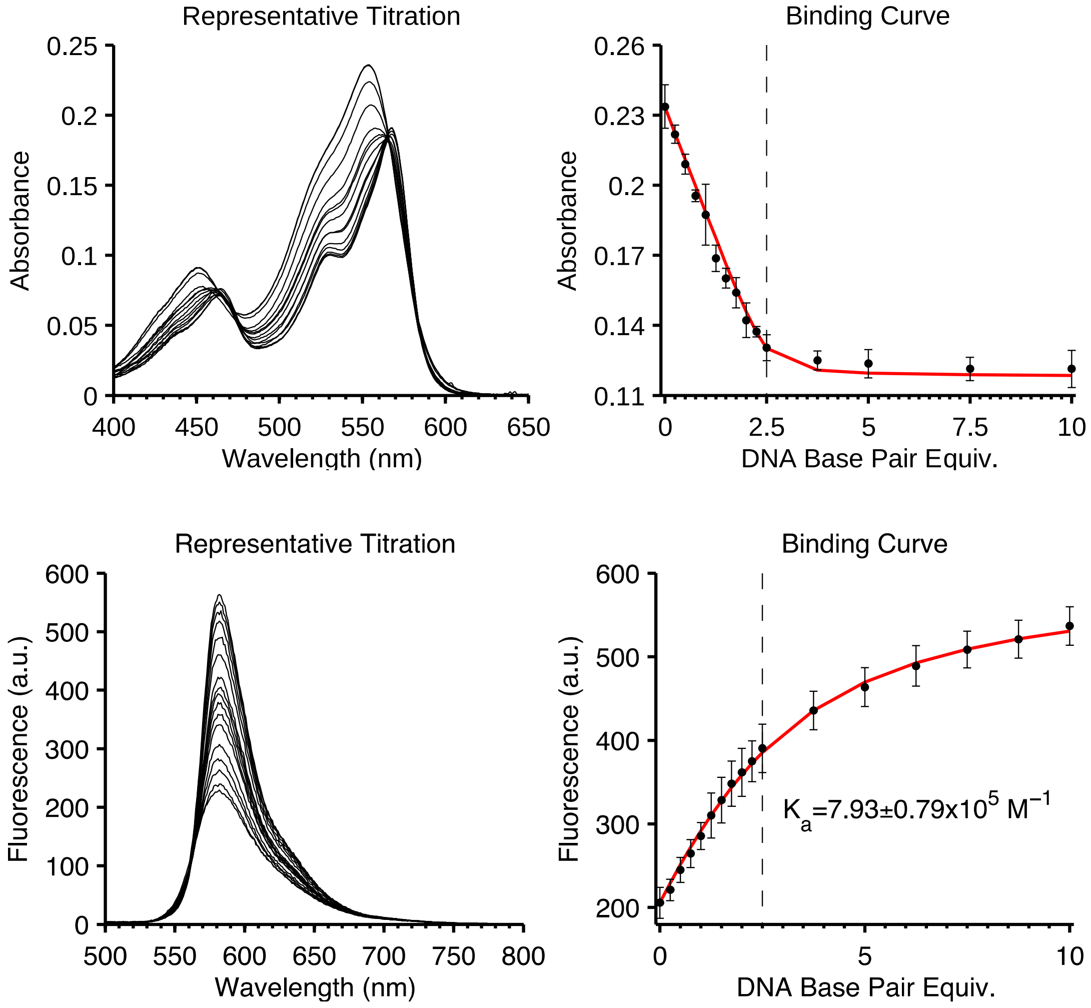

ii. ds17

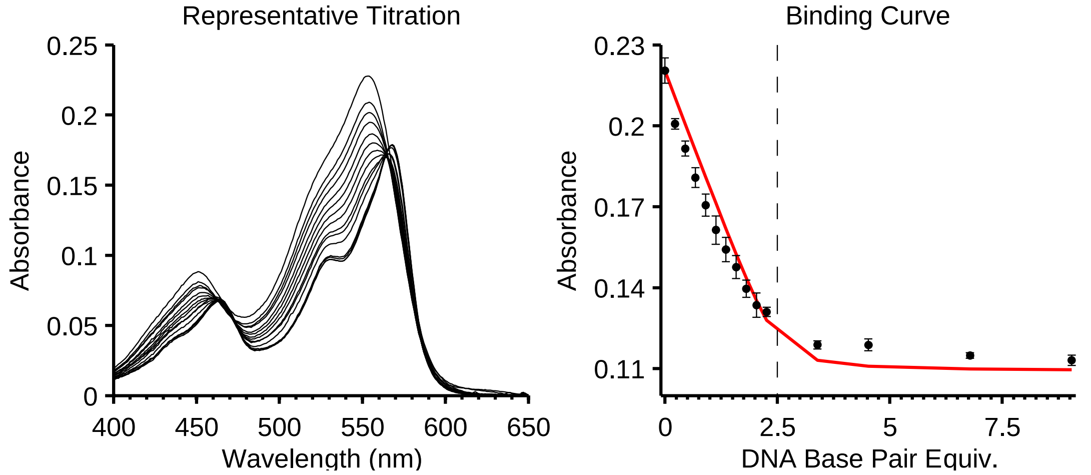

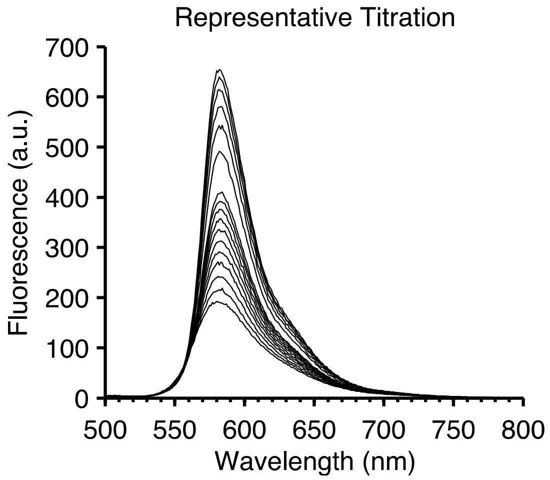
Representative Titration
Fluorescence (a.u.)
Wavelength (nm)

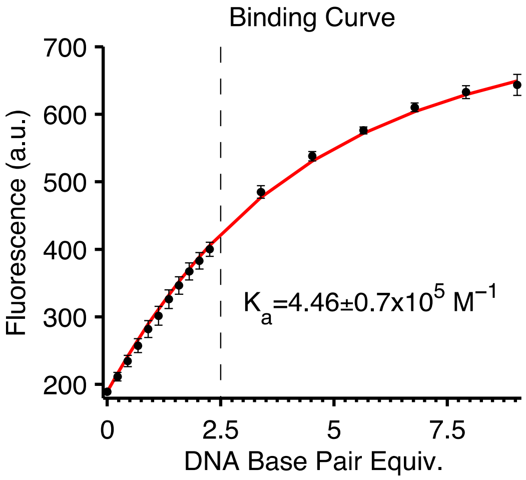
Binding Curve
Fluorescence (a.u.)
DNA Base Pair Equiv.
K_a=4.46±0.7x10^5 M^{-1}

iii. ss17

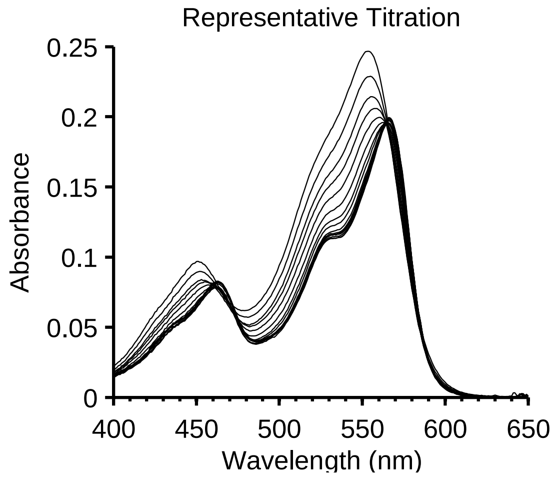
Representative Titration
Absorbance
Wavelength (nm)

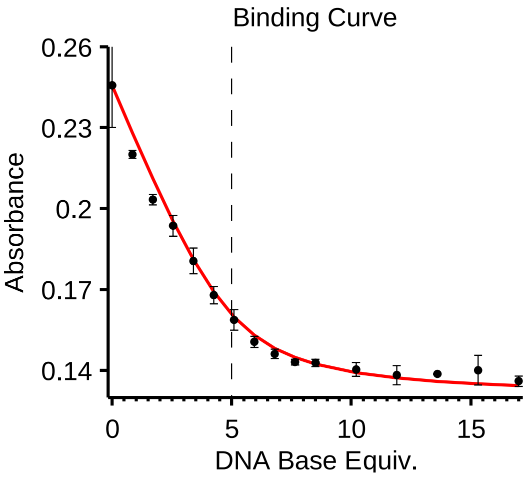
Binding Curve
Absorbance
DNA Base Equiv.

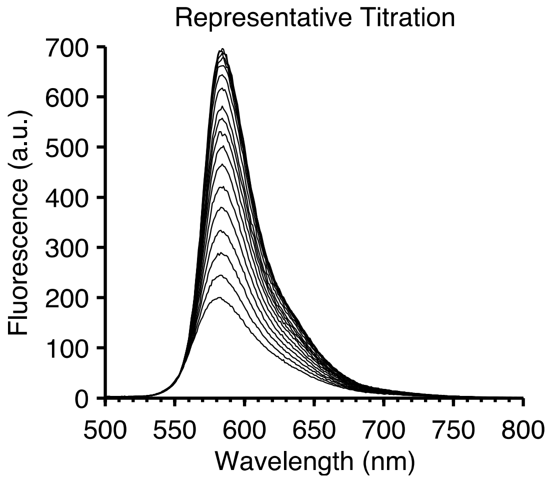
Representative Titration
Fluorescence (a.u.)
Wavelength (nm)

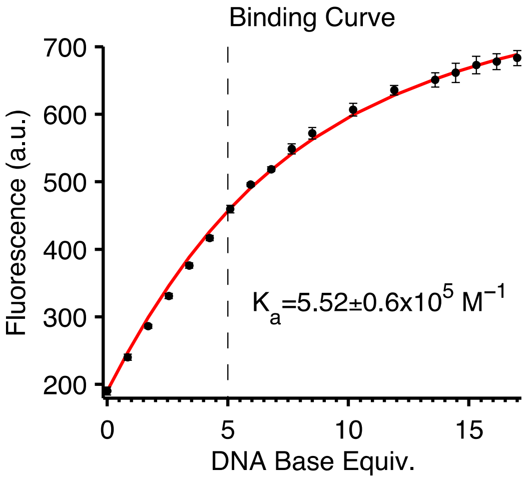
Binding Curve
Fluorescence (a.u.)
DNA Base Equiv.
K_a=5.52±0.6x10^5 M^{-1}

iv. TBA

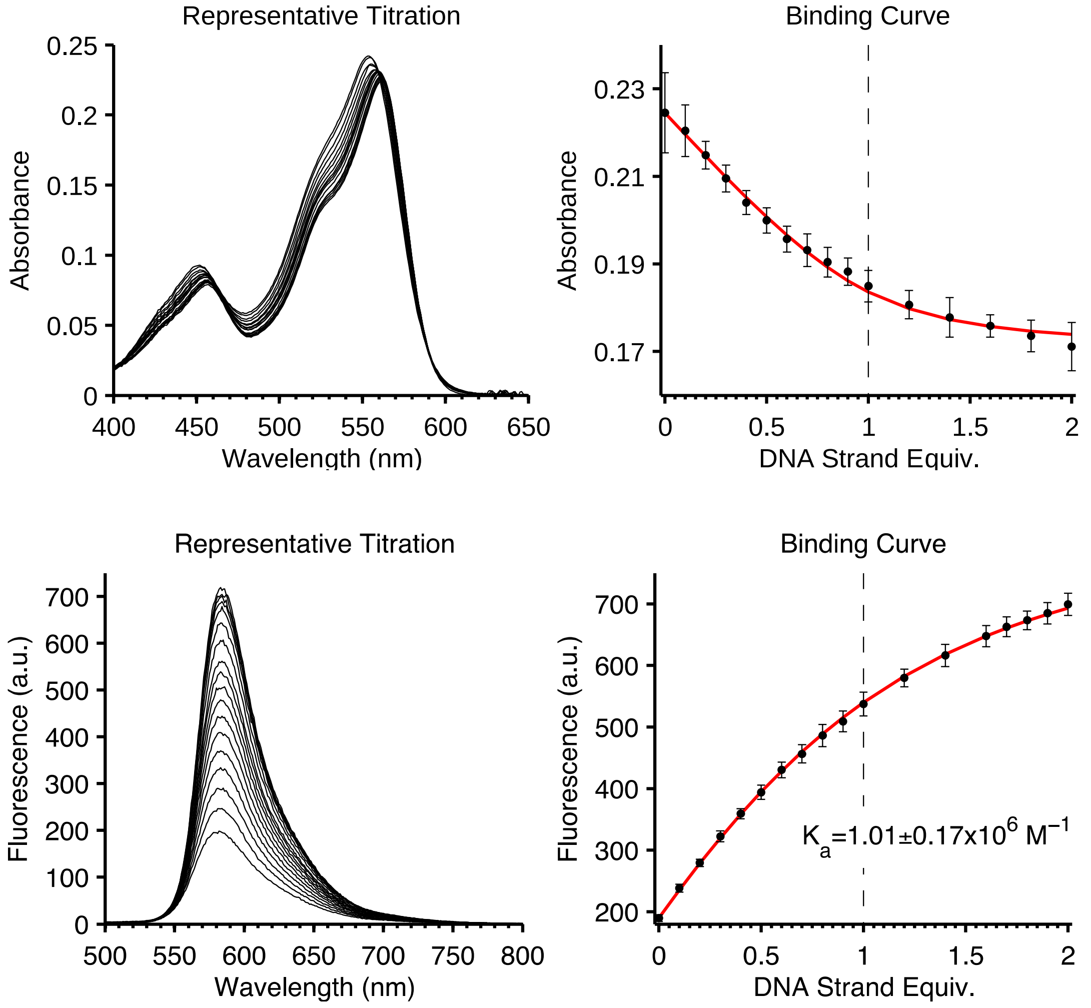

v. myc2345

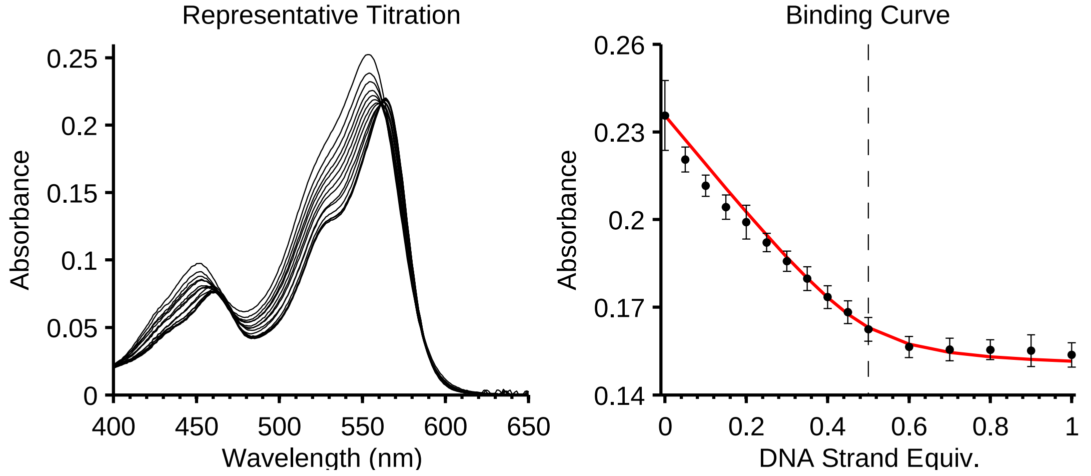

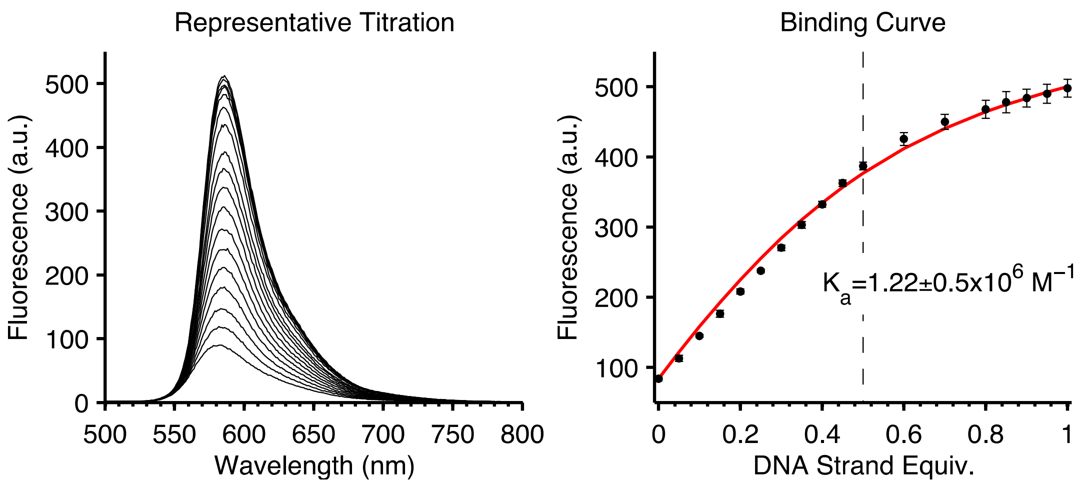

vi. PDGFA-48mer

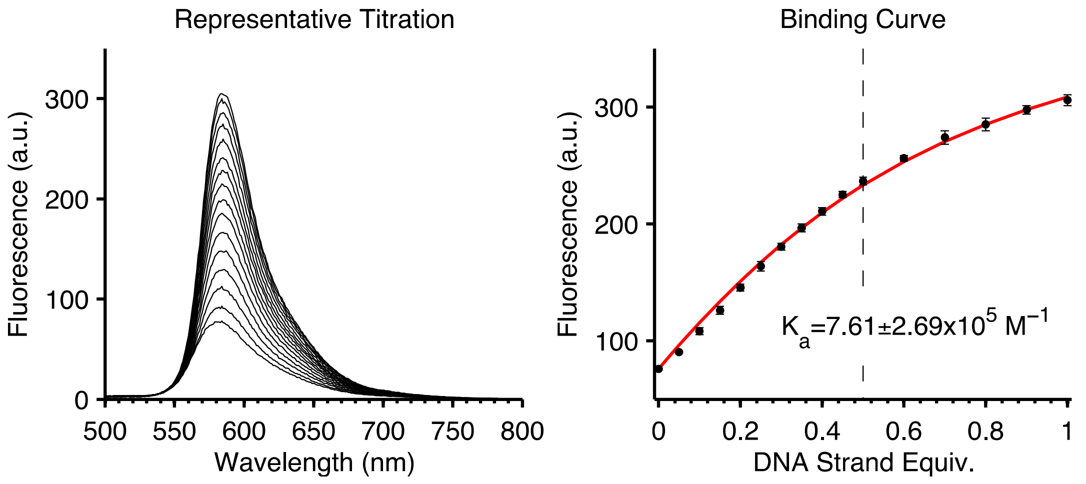

Supplementary Figure 7. Absorbance and emission titrations for **DAOTA-M2** with various DNA topologies. Error bars represent the standard deviation of three independent repeats. The average fitting is shown with no error bars (red) for clarity. Dashed lines indicate stoichiometry. No absorption titrations for PDGF-A were performed due to the DNA annealing concentration being lower than the compound's concentration used for titration.

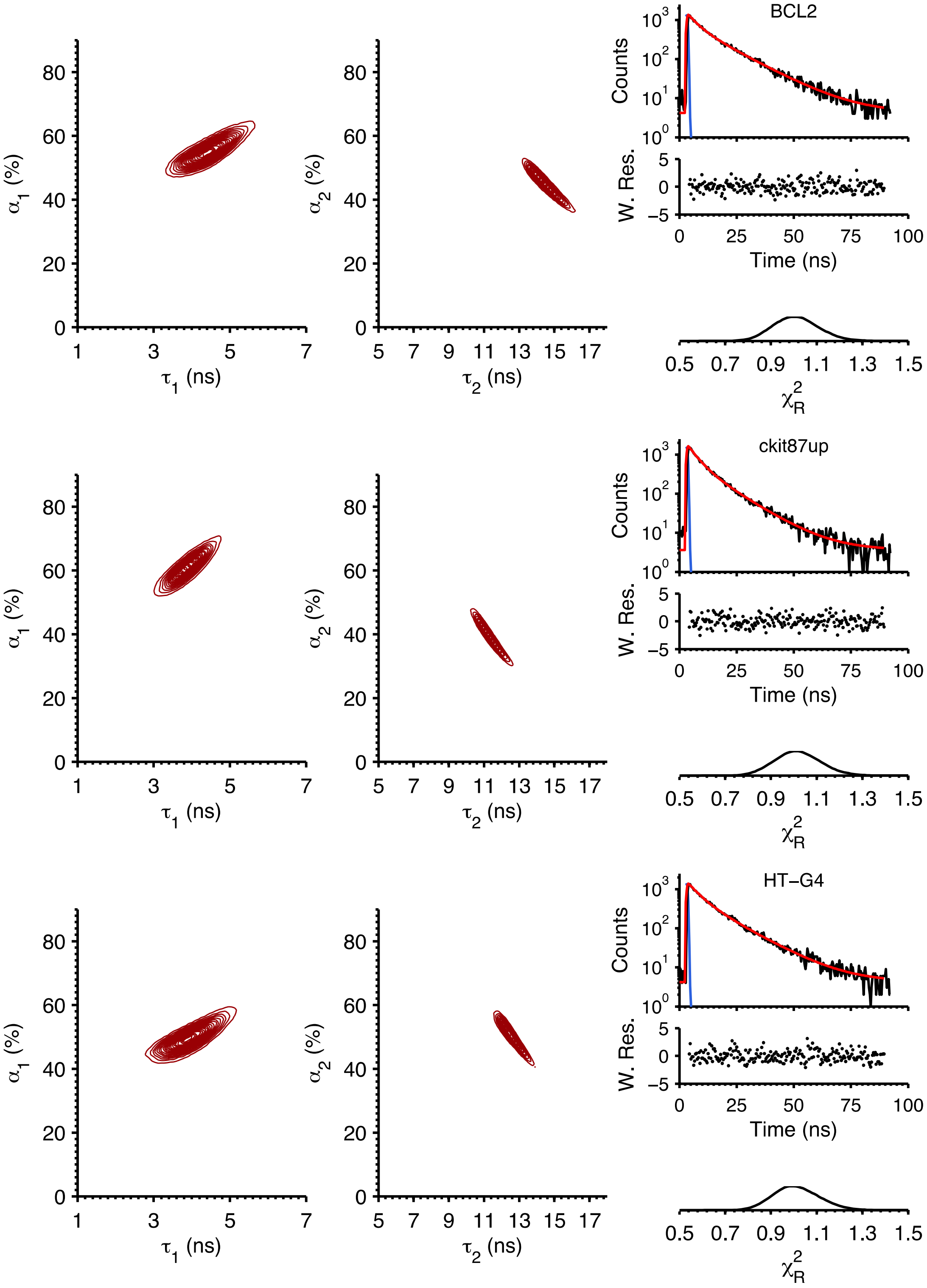
BCL2
ckit87up
HT−G4
Counts
W. Res.
Time (ns)
χ^2_R
α_1 (%)
α_2 (%)
τ_1 (ns)
τ_2 (ns)

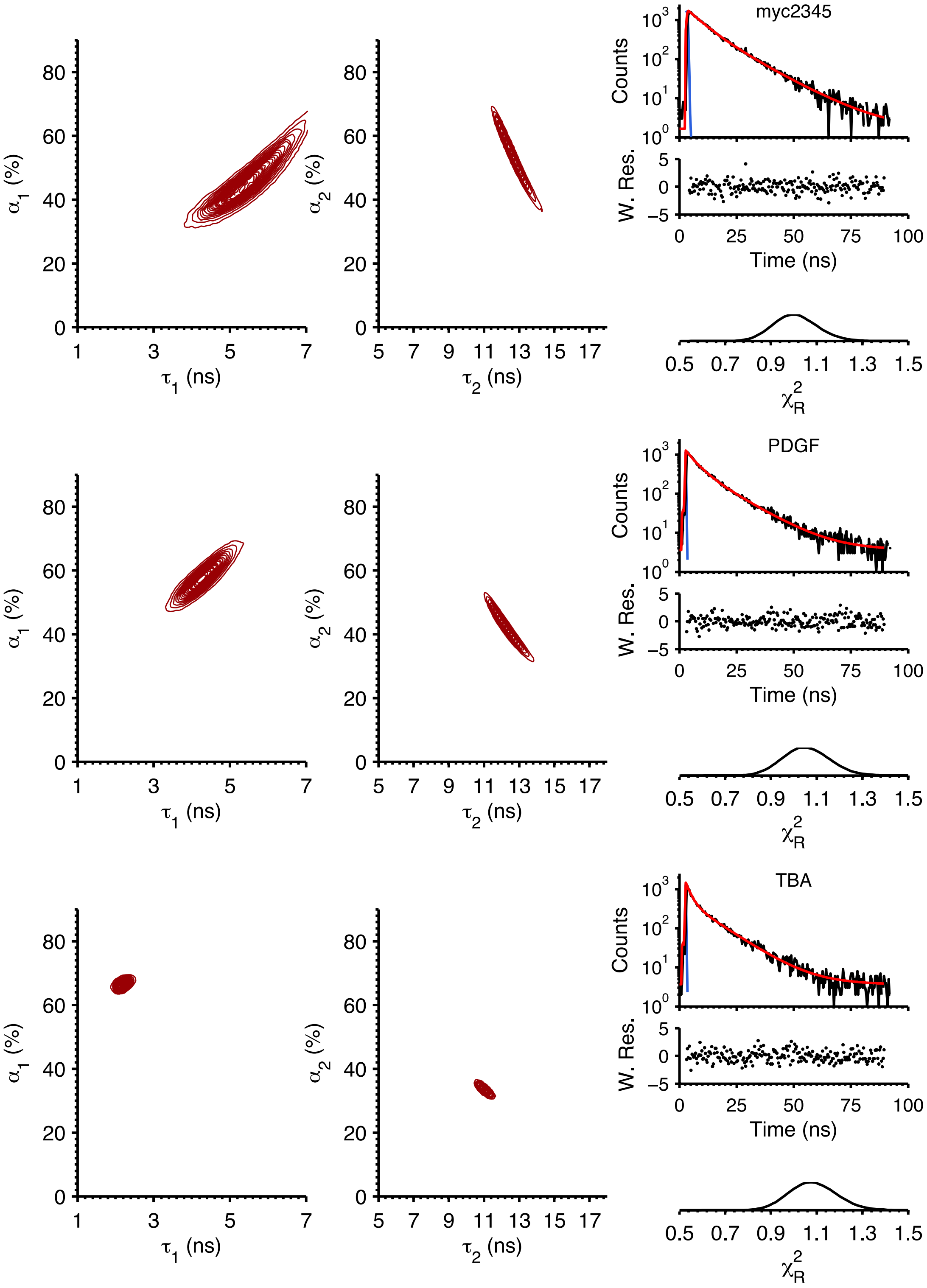
myc2345
PDGF
TBA
α1 (%)
α2 (%)
τ1 (ns)
τ2 (ns)
Counts
W. Res.
Time (ns)
χ2R
0
20
40
60
80
1
3
5
7
9
11
13
15
17
25
50
75
100
−5
0.5
0.7
0.9
1.1
1.3
1.5
10^0
10^1
10^2
10^3

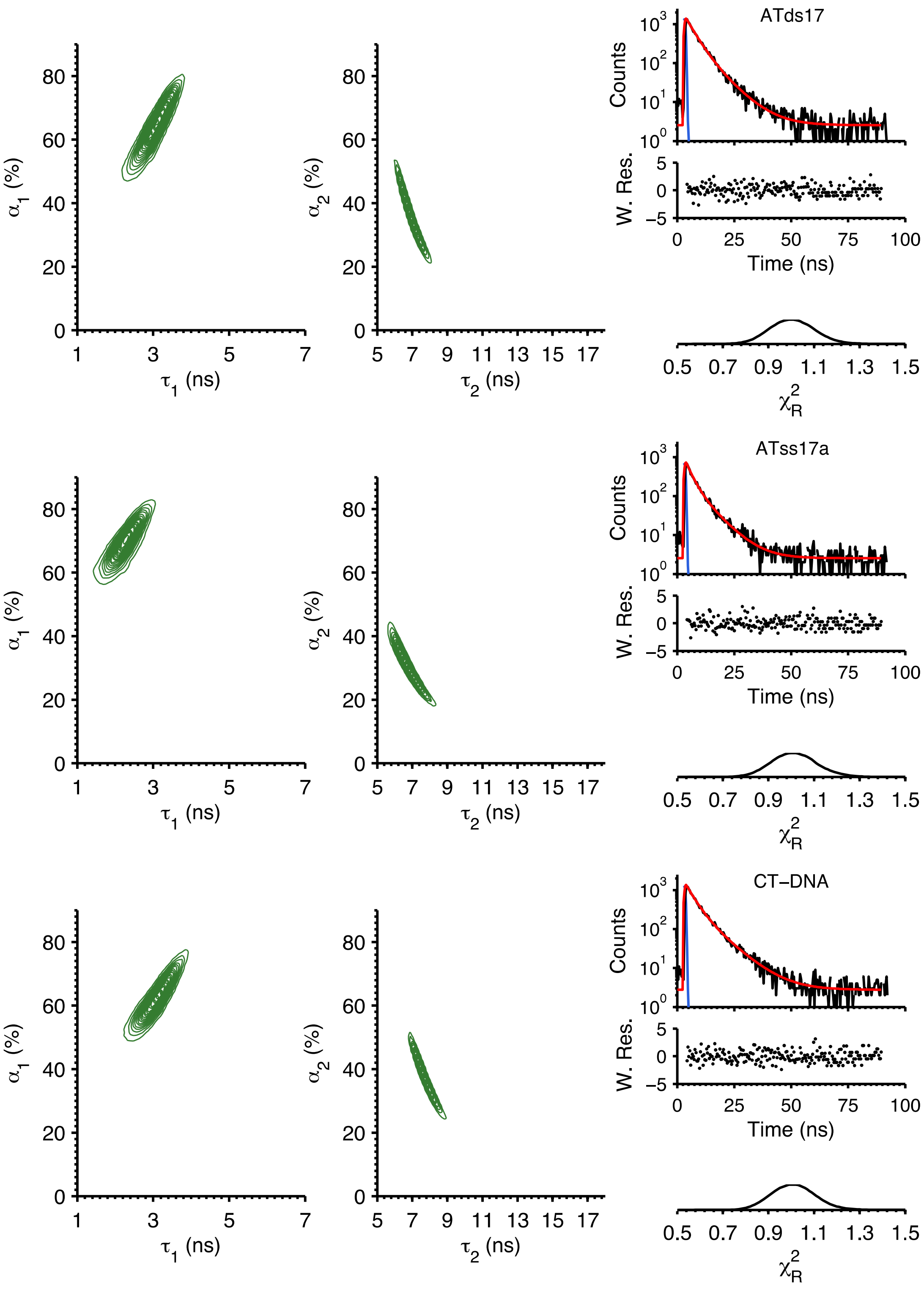
ATds17
ATss17a
CT–DNA
Counts
W. Res.
Time (ns)
α1 (%)
α2 (%)
τ1 (ns)
τ2 (ns)
χ2R

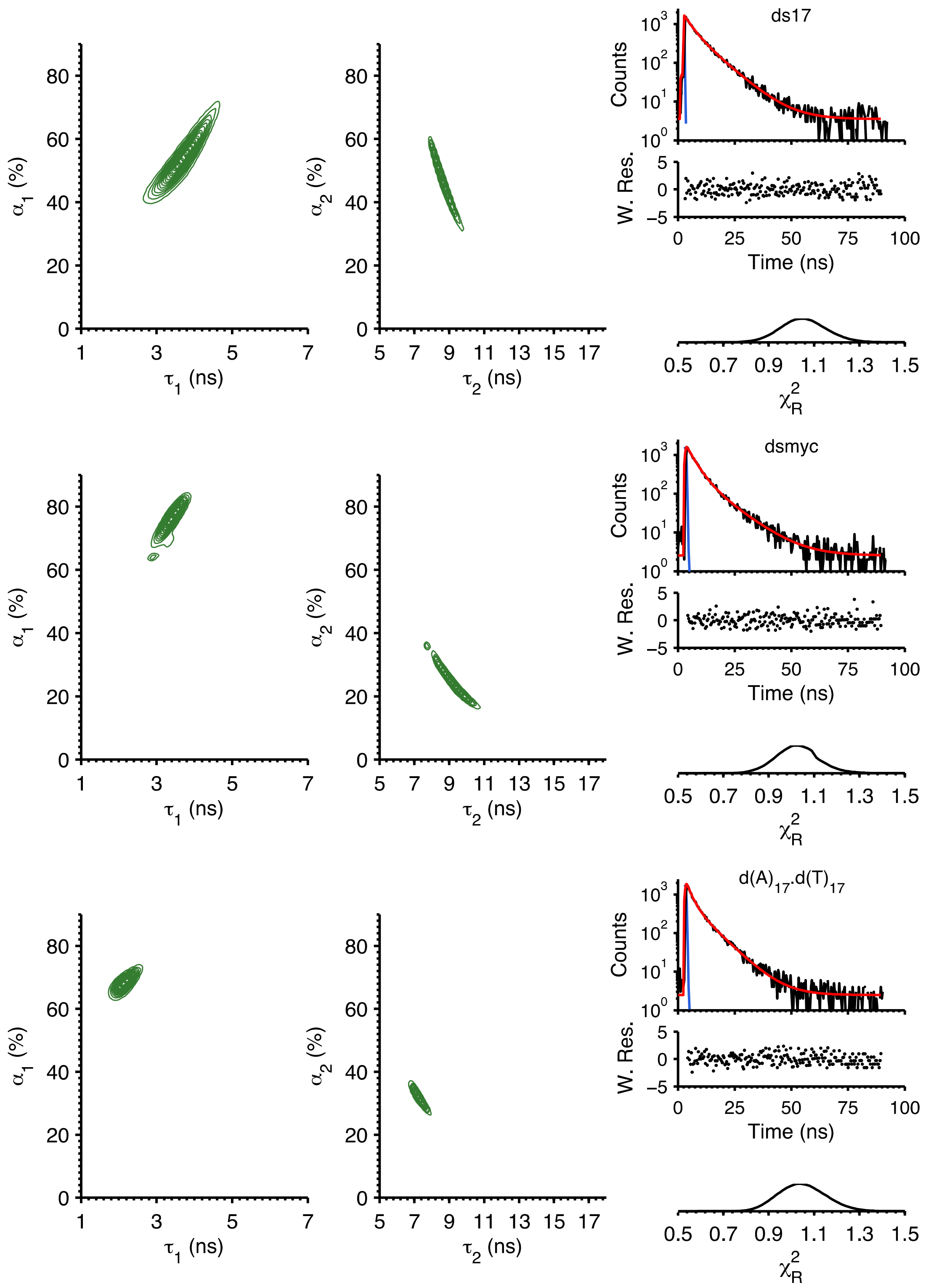
ds17
dsmyc
d(A)17.d(T)17
α1 (%)
α2 (%)
τ1 (ns)
τ2 (ns)
Counts
W. Res.
Time (ns)
χ2R

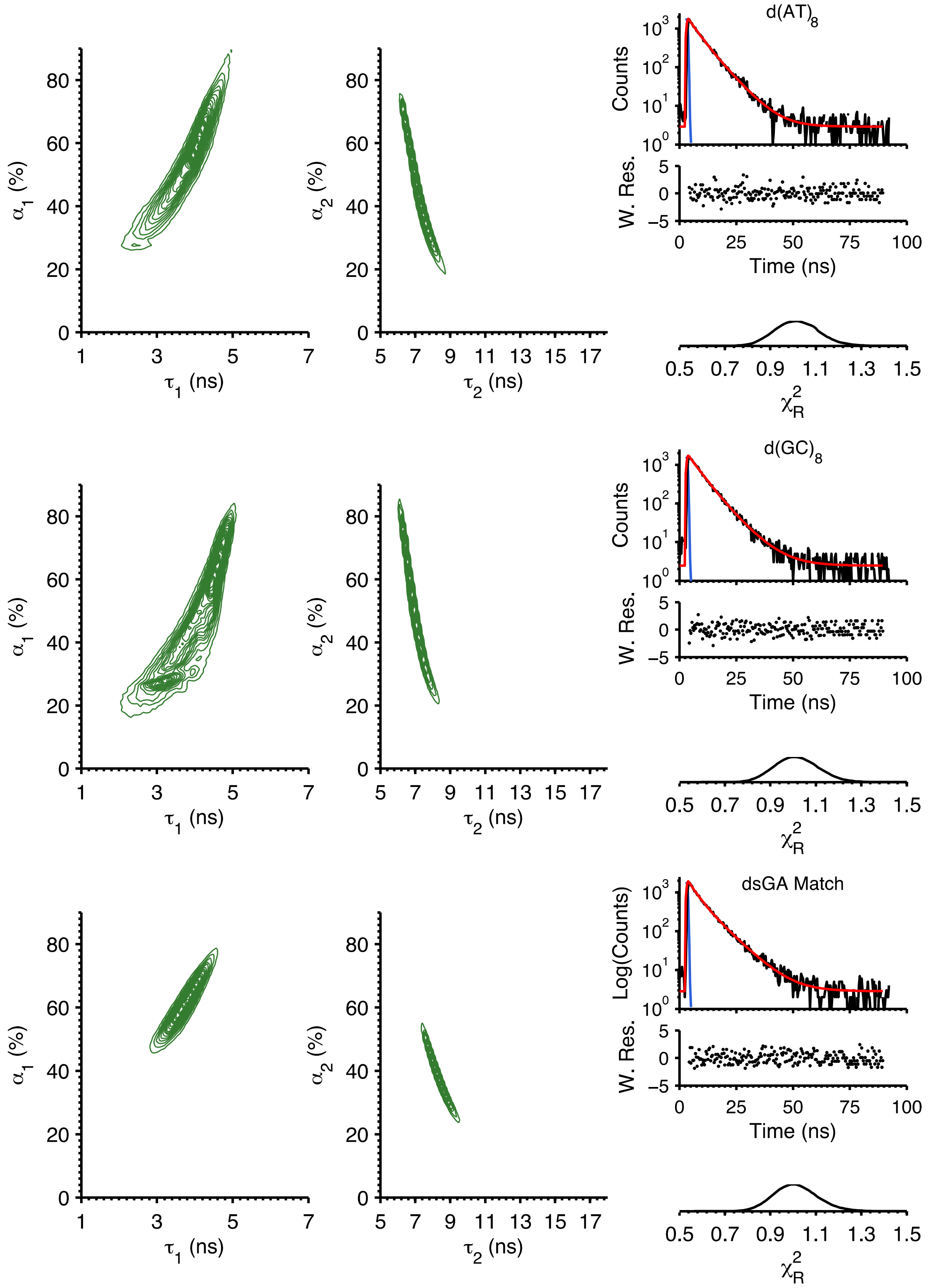

d(AT)8
d(GC)8
dsGA Match
α1 (%)
α2 (%)
τ1 (ns)
τ2 (ns)
Counts
Log(Counts)
W. Res.
Time (ns)
χR2

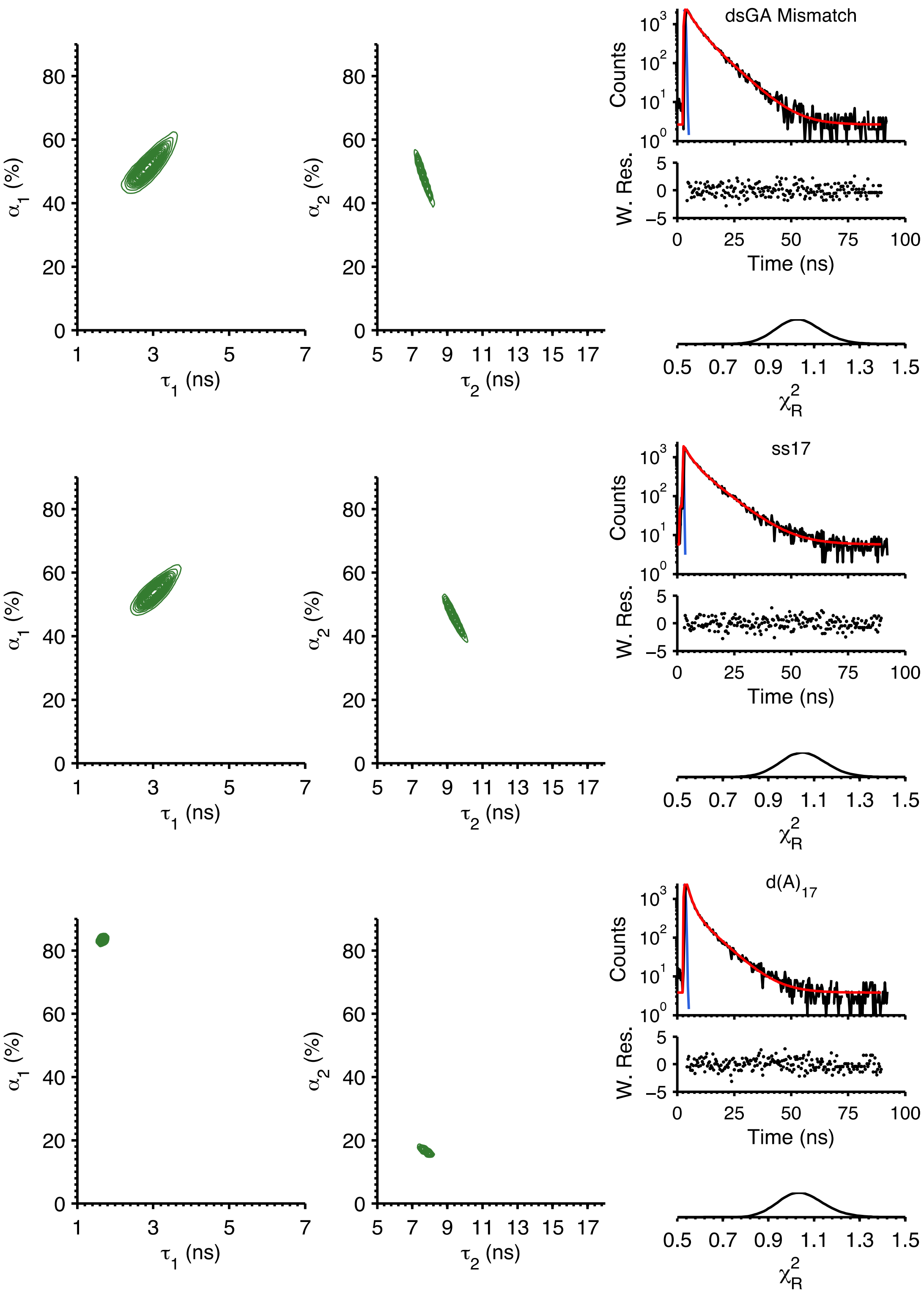
dsGA Mismatch
ss17
d(A)17
Counts
W. Res.
Time (ns)
χ^2_R
α_1 (%)
α_2 (%)
τ_1 (ns)
τ_2 (ns)

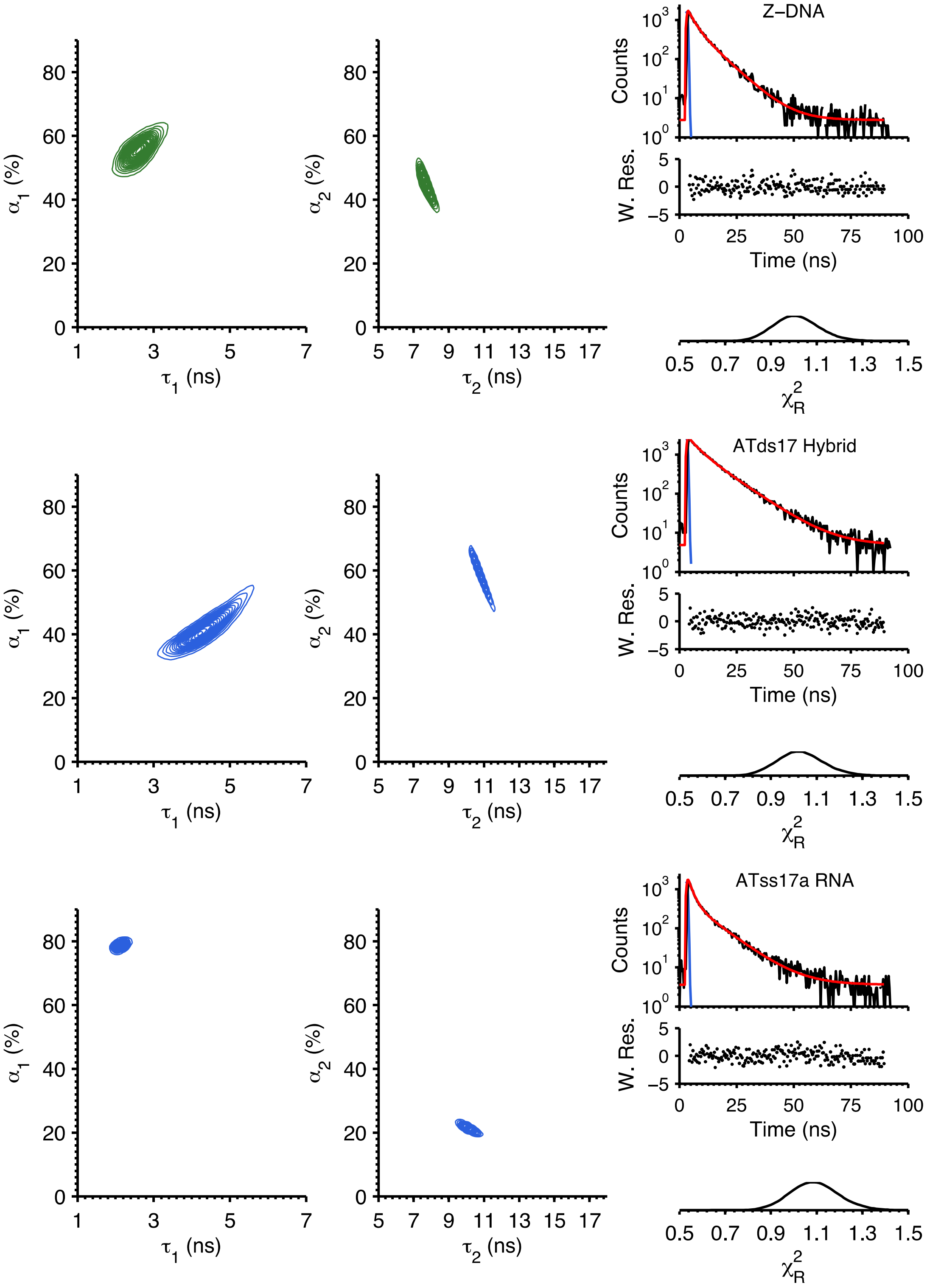
Z–DNA
ATds17 Hybrid
ATss17a RNA
Counts
W. Res.
Time (ns)
χ^2_R
α_1 (%)
α_2 (%)
τ_1 (ns)
τ_2 (ns)

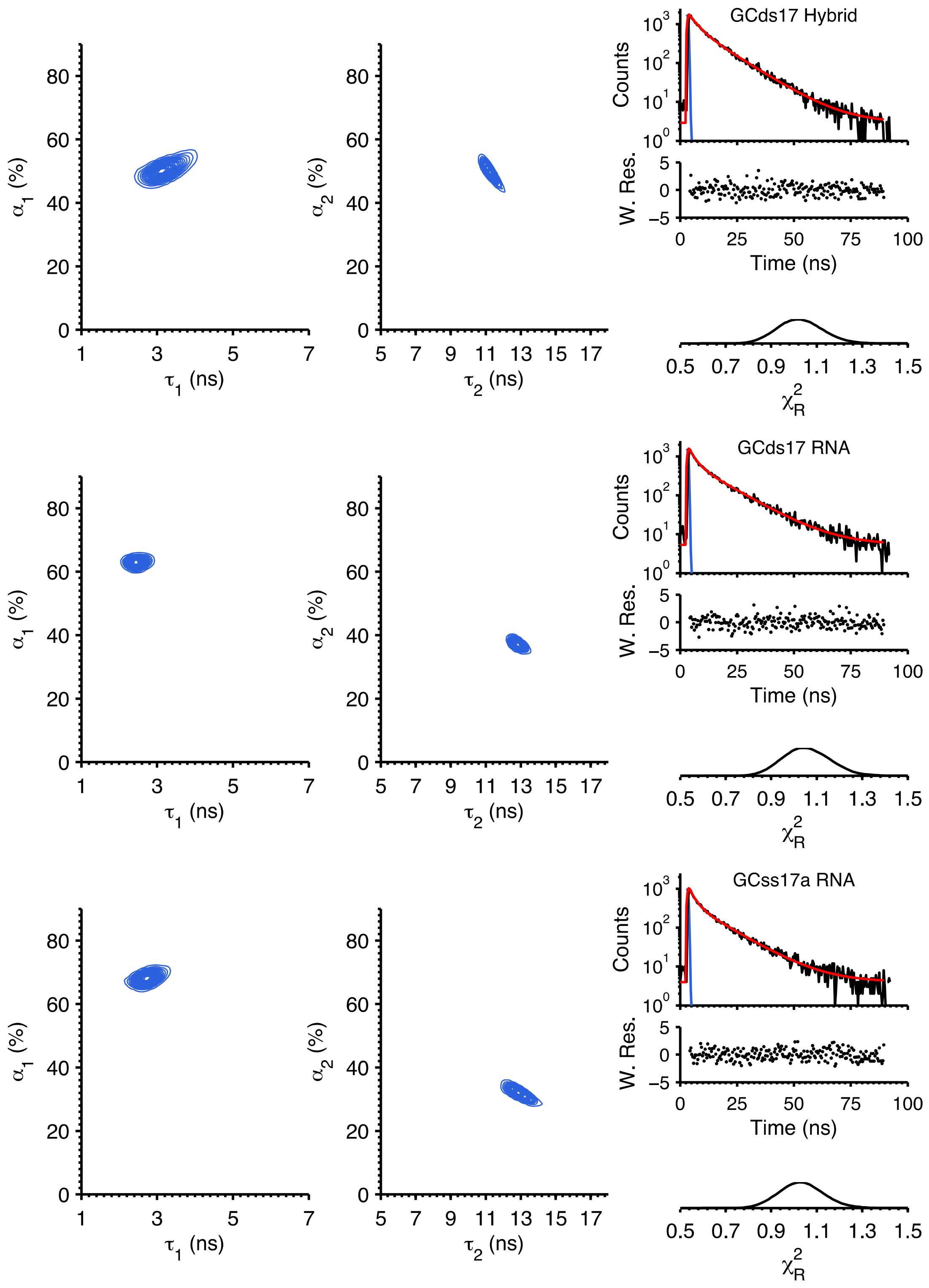

GCds17 Hybrid
GCds17 RNA
GCss17a RNA
Counts
W. Res.
Time (ns)
χ^2_R
α_1 (%)
α_2 (%)
τ_1 (ns)
τ_2 (ns)

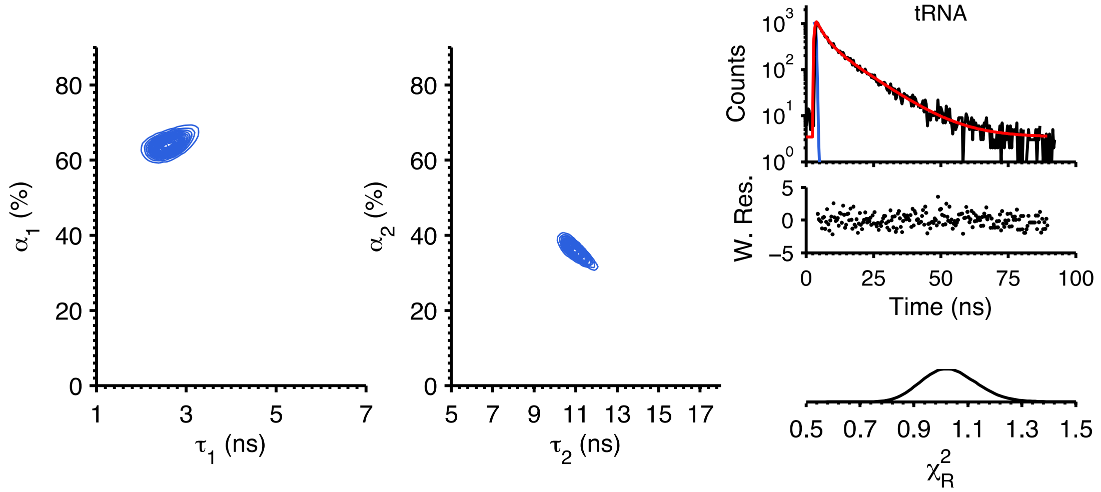

Supplementary Figure 8. Individual nucleic acid model contour maps for fluorescence lifetime and the corresponding amplitude for each pixel of the FLIM image (512 × 512), representative decay traces and probability density histograms of χ^2_R. Fittings are shown in red and the instrument response function in blue. Contour maps are colour coded depending upon the model is part of the G-quadruplex (red), DNA (green) or RNA (blue) groups.

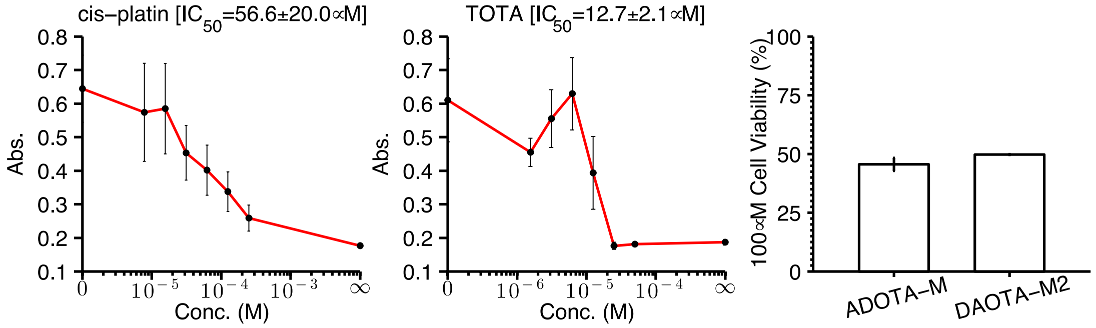

Supplementary Figure 9. The absolute IC_{50} of cis-platin (control), **TOTA**, **ADOTA-M** and **DAOTA-M2** in the U2OS cell line at 24 h. The IC_0 and IC_{100} values are represented by 0 and ∞ respectively on the x-axis. For **ADOTA-M** and **DAOTA-M2**, cell viability was reduced to ~50% or above at 100 uM. Error bars represent the standard deviation of three independent repeats.

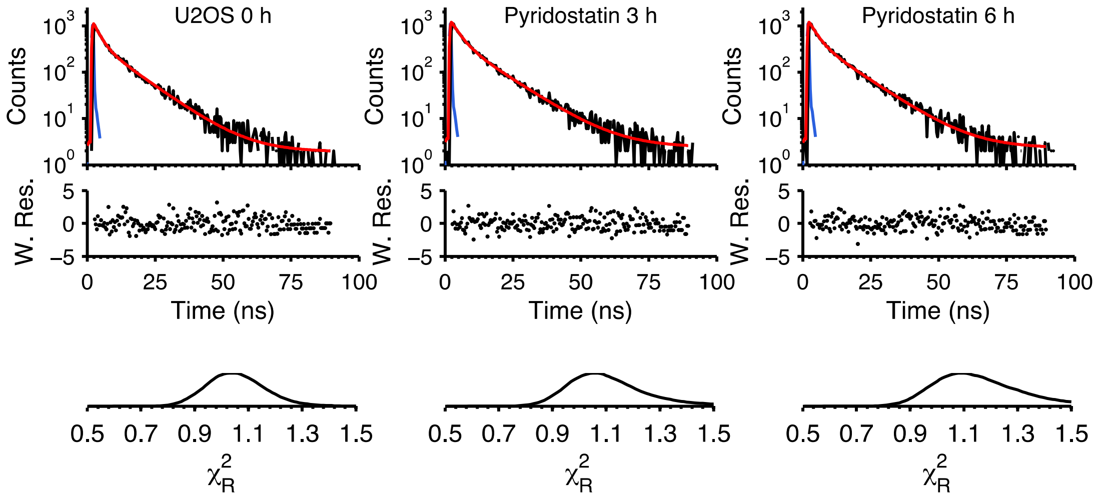

Supplementary Figure 10. Representative cellular FLIM decay traces and probability density histograms of χ_R^2 for Fig. 5 and Supplementary Fig. 11 data. Fittings are shown in red and the instrument response function in blue.

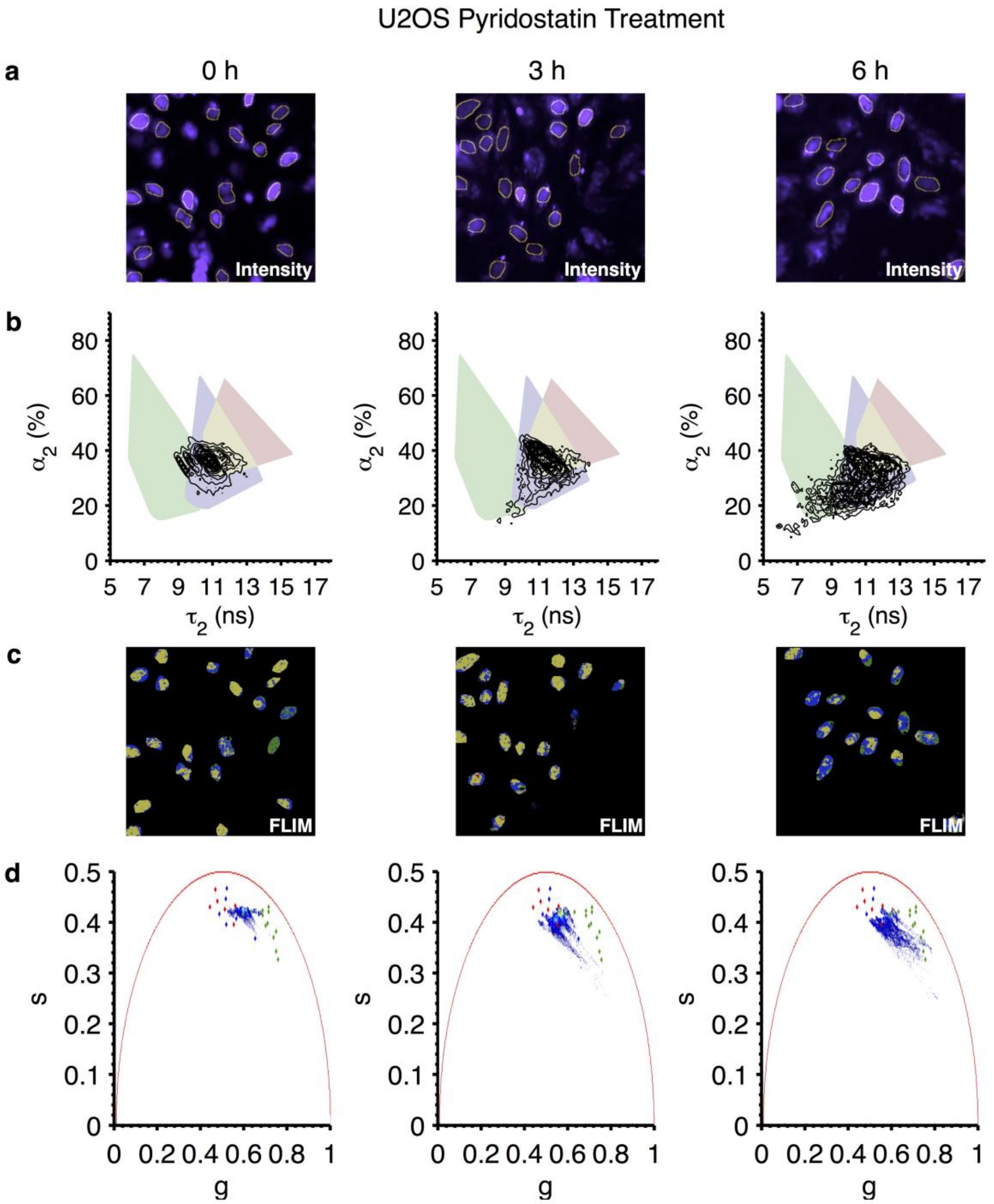

Supplementary Figure 11. Masked FLIM visualisation of U2OS cells incubated with **DAOTA-M2** (20 µM, 24 h) and subsequently treated with G-quadruplex selective ligand pyridostatin (10 µM) over time (0 – 6 h). (**a**) illustrates the mask (yellow) applied to intensity (purple) images. Only pixels inside the mask were analysed to reduce cytoplasmic signals. (**b**) shows cellular τ_2 and α_2 plotted in the form of a contour map and superimposed upon *in vitro* nucleic acid models boundaries for G-quadruplexes (red), DNA (green), RNA (blue) or overlapping RNA / G-quadruplexes (yellow). The difference between each contour level is 5 % of the maximum bin frequency for the cellular data and the minimum contour contains 95 % of the data relative to the maximum bin frequency. (**c**) presents images colour coded based on the location of the cellular pixel τ_2 and α_2 with respect to the *in vitro* models boundaries.

(**d**) depicts phasor transformed cellular fluorescence decays as a heat map of pixels (red – highest frequency, blue – lowest frequency) superimposed upon *in vitro* colour coded nucleic acid models.

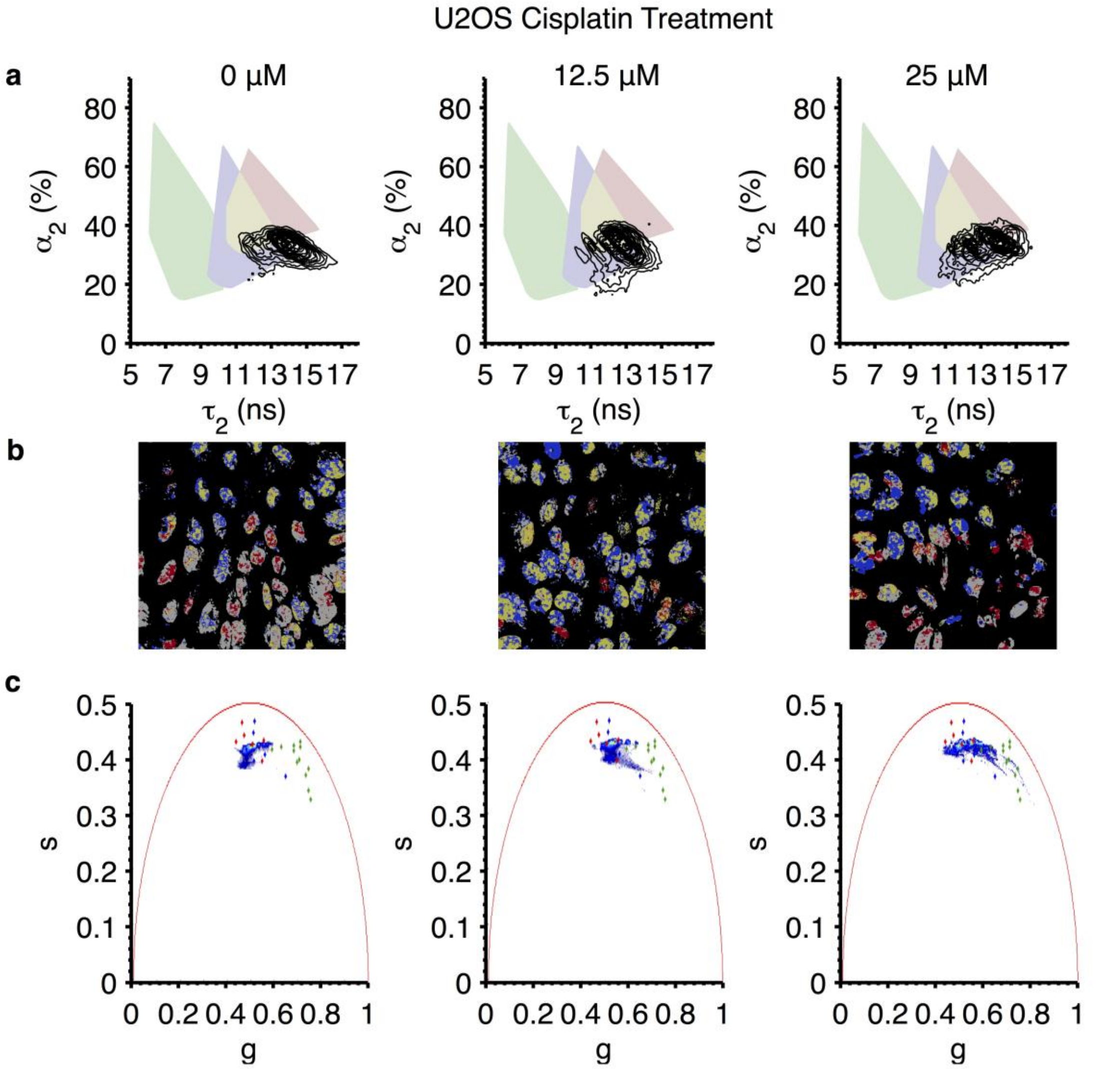

Supplementary Figure 12. FLIM visualisation of U2OS cells co-incubated with **DAOTA-M2** (20 µM, 24 h) and non-specific DNA damaging agent cisplatin (12.5 / 25 µM, 24 h). It should be noted that for 25 µM cisplatin treatments there was some reduction in cell viability prior to imaging. (**a**) shows cellular τ_2 and α_2 plotted in the form of a contour map and superimposed upon *in vitro* nucleic acid models boundaries for G-quadruplexes (red), DNA (green), RNA (blue) or overlapping RNA / G-quadruplexes (yellow). The difference between each contour level is 5 % of the maximum bin frequency for the cellular data and the minimum contour contains 95 % of the data relative to the maximum bin frequency. (**b**) presents images colour coded based on the location of the cellular pixel τ_2 and α_2 with respect to the *in vitro* models boundaries. (**c**) depicts phasor transformed cellular fluorescence decays as a heat map of pixels (red – highest frequency, blue – lowest frequency) superimposed upon *in vitro* colour coded nucleic acid models. See Supplementary Fig. 13 for χ_r^2, representative decay traces and fits for all data.

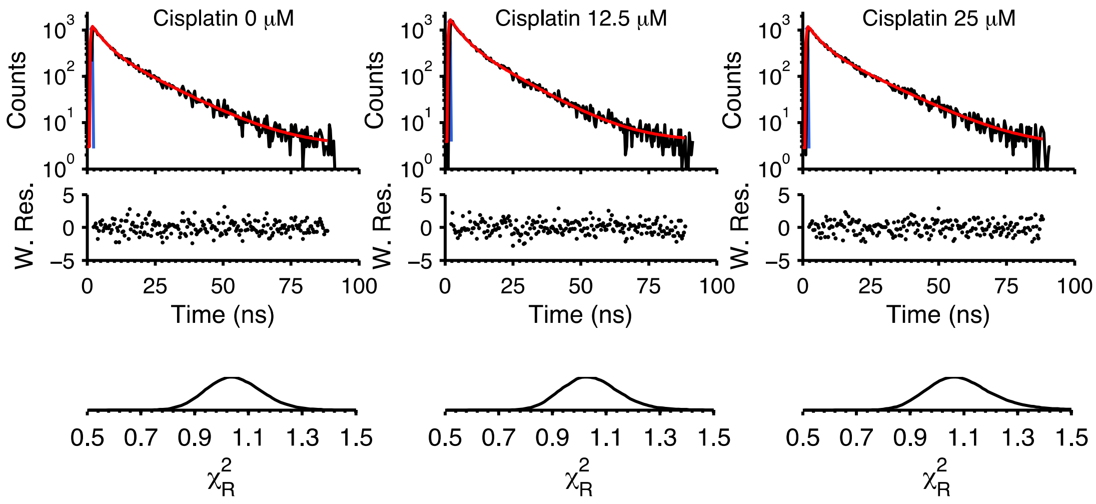

Supplementary Figure 13. Representative cellular FLIM decay traces and probability density histograms of χ_R^2 for Supplementary Fig. 12 data. Fittings are shown in red and the instrument response function in blue.

Supplementary Methods

General Synthetic Procedures. All chemicals were used as purchased from commercial sources unless stated otherwise. Solvents were distilled over an appropriate drying agent and degassed prior to use. ^{1}H and ^{13}C NMR spectra were recorded using a 400 MHz and 500 MHz Bruker Avance Ultrashield NMR spectrometer at 296 K respectively. Chemical shifts are referenced to residual deuterated solvent. Mass spectra were obtained using electrospray ionisation (ES) by Mrs. L. Haigh (Imperial College London) on a Bruker Daltronics Esquire 3000 spectrometer. Microanalyses of the compounds were performed by Mr. A. Dickerson (Cambridge University).

*Tris(2,6-dimethoxyphenyl)methylium tetrafluoroborate (**1**).* A modified procedure to that reported by B. W. Laursen *et al* was used.[1] *N,N,N',N'*-tetramethylethylenediamine (0.1 mL, 1 mmol) was added to a solution of *n*-butyllithium (12 mL, 2.0 M, 24 mmol) in cyclohexane under a nitrogen atmosphere. The mixture was then cooled to 0 ^{o}C before the addition of 1,3-dimethoxybenzene (3.0 mL, 23 mmol). After warming to room temperature, dry diethyl ether (10 mL) was added to the white suspension followed by anhydrous diethyl carbonate (0.9 mL, 7 mmol). The solution turned a deep orange after stirring for 24 h. Addition of water (50 mL) and then ethyl acetate (50 mL) was followed by washing of the organic phase with more water (2 × 50 mL). The organic phase was dried over magnesium sulfate, filtered and concentrated *in vacuo* to yield an orange oil / solid. This mixture was then suspended in diethyl ether (30 mL) before tetrafluoroboric acid in diethyl ether (1.9 mL, 14 mmol) was added. Filtration of the violet suspension and washing with diethyl ether (3 x 30 mL) gave a black powder (crude **1**) which was used without further purification. Yield: 3.06 g, 5.99 mmol, 86%; ^{1}H NMR (400 MHz, CD_3CN): δ_H 7.62 (t, 3 H, $^{3}J_{HH}$ = 8.4 Hz, Ar*H* ortho to OMe), 6.59 (d, 6 H, $^{3}J_{HH}$ = 8.4 Hz, Ar*H* meta to OMe), 3.54 (s, 18H, OCH_3); ^{13}C NMR (101 MHz, CD_3CN): δ_c 163.7, 143.2, 126.4, 106.1, 57.4; ES^+-MS m/z 423.2 (M^+-BF_4^- where M=$C_{25}H_{27}O_6(BF_4)$, 100).

*4,8,12-trioxadibenzo[cd,mn]pyren-12-ium tetrafluoroborate (**TOTA**).* A modified procedure to that reported by J. C. Martin et al was used.[2] Pyridine hydrogen chloride (5.00 g) was mixed with **1** (0.10 g, 0.20 mmol) and dried under vacuum. The solid mixture was then stirred and heated to 200 ^{o}C. After 1 h, the deep red solution was cooled to room temperature and hydrochloric acid (0.1 M, 50 mL) was added. The red insoluble material was removed by filtration and the bright yellow filtrate was basified (pH = 14) with sodium hydroxide pellets. The white precipitate was recovered by filtration and thoroughly washed with a solution of sodium hydroxide (0.1 M). The solid was then dissolved in diethyl ether (50 mL) and filtered. Addition of tetrafluoroboric acid in diethyl ether (0.10 mL, 0.73 mmol) to the colourless filtrate and filtration of the resultant suspension yielded **TOTA** as a yellow powder. Yield: 27 mg, 0.07 mmol, 37%; Elem. Anal. $C_{19}H_9O_3(BF_4)$ Calc. %C 61.33, %H 2.44,

Found %C 61.09, %H 2.47; ^{1}H NMR (400 MHz, CD_3CN): δ_H 8.47 (t, 3 H, $^3J_{HH}$ = 8.4 Hz, Ar H-2, H-6 and H-10), 7.79 (d, 6 H, $^3J_{HH}$ = 8.4 Hz, Ar H-1, H-3, H-5, H-7, H-9 and H-11); ^{13}C NMR (400 MHz, CD_3CN): δ_c 154.6, 146.6, 144.6, 113.5, 107.2; MS-ES$^+$ m/z 285.1 (M^+-BF_4^- where M=$C_{19}H_9O_3(BF_4)$, 100).

*9-(2,6-dimethoxyphenyl)-1,8-dimethoxy-10-(2-morpholinoethyl)-acridin-10-ium hexafluorophosphate (**2**).* 2-morpholinoethanamine (78 µL, 0.59 mmol) was added to a solution of **1** (0.20 g, 0.39 mmol) in 1-methyl-2-pyrrolidinone (1.4 mL). The deep orange / red solution formed was left to stir at room temperature for 2 h before a solution of ammonium hexafluorophosphate (0.13 g, 0.80 mmol) in water (10 mL) was added. The red precipitate formed was recovered by filtration, thoroughly washed with water and then diethyl ether. After allowing drying, the solid was repeatedly re-crystallised from dichloromethane and *n*-pentane. Red needles of **2** were obtained after filtration. Yield: 87 mg, 0.13 mmol, 55%; Elem. Anal. $C_{29}H_{33}N_2O_5(PF_6)$ Calc. %C 54.89, %H 5.24, %N 4.41, Found %C 54.77, %H 5.17, %N 4.22%; ^{1}H NMR (400 MHz, CD_3CN): δ_H 8.19 (dd, 2 H, $^3J_{HH}$ = 9.3 and 8.1 Hz, Acridinium H-3 and H-6), 7.95 (d, 2H, $^3J_{HH}$ = 9.3 Hz, Acridinium H-4 and H-5), 7.43 (t, 1 H, $^3J_{HH}$ = 8.3 Hz, Ar H-4), 7.09 (d, 2 H, $^3J_{HH}$ = 8.1 Hz, Acridinium H-2 and H-7), 6.77 (d, 2 H, $^3J_{HH}$ = 8.3 Hz, Ar H-3 and H-5), 5.24 (t, 2 H, $^3J_{HH}$ = 6.6 Hz, $N_{Ar}CH_2$), 3.62 – 3.57 (m, 4 H, OCH_2), 3.54 (s, 6 H, OCH_3), 3.53 (s, 6 H, OCH_3), 3.10 (t, 2 H, $^3J_{HH}$ = 6.6 Hz, $NC_{alkyl}H_2$), 2.62 – 2.54 (m, 4 H, $NC_{ring}H_2$); ^{13}C NMR (400 MHz, CD_3CN): δ_C 161.5, 158.4, 156.7, 143.1, 140.8, 130.4, 120.9, 120.6, 110.7, 107.5, 104.7, 67.5, 57.8, 56.6, 56.4, 54.8, 51.8; ES(+)-MS m/z $C_{29}H_{33}N_2O_5$ Calc. 489.2389, Found 489.2398 a.m.u.

*12-(2-morpholinoethyl)-4,8-dioxa-12-azadibenzo[cd,mn]-pyren-12-ium hexafluorophosphate (**ADOTA-M**).* Pyridine hydrogen chloride (10.00 g) was mixed with **2** (0.20 g, 0.31 mmol) and dried under vacuum before the solid was stirred and heated to 200 ^{o}C for 1 h with shielding from light. The deep red solution was cooled to room temperature and acetonitrile (100 mL) was added. The crude red solid was recovered by centrifugation at 4,000 rpm for 2 min and washed with acetonitrile (2 × 50 mL) and then diethyl ether (2 × 20 mL). Anion exchange was performed by dissolving the solid in an aqueous solution of sodium hexapfluorophosphate (0.1 M, 5 mL), and extracting with dichloromethane (10 mL). The pH of the aqueous layer during this process was adjusted to 14. The addition of an equal volume of ethyl acetate and to organic phase and slow evaporation of this solution when shielded from light gave red plates of **ADOTA-M**. These crystals were dissolved in dichloromethane and the crystallisation process repeated to give **ADOTA-M**. Yield: 19 mg, 0.04 mmol, 11%; Elem. Anal. $C_{25}H_{21}N_2O_3(PF_6).0.5H_2O$ Calc. %C 54.45; %H 4.02, %N 5.08, Found %C 54.26, %H 3.77, %N 4.99; ^{1}H NMR (400 MHz, CD_3CN): δ_H 8.34 (dd, 2 H, $^3J_{HH}$ = 8.9 and 8.5 Hz, Ar H-2 and H-10), 8.12 (t, 1 H, $^3J_{HH}$ = 8.5 Hz, Ar H-6), 7.85 (d, 2 H, $^3J_{HH}$ = 8.9 Hz, Ar H-1 and H-11), 7.57 (d, 2 H, $^3J_{HH}$ = 8.3 Hz, Ar*H*), 7.50 (d, 2 H, $^3J_{HH}$ = 8.5 Hz, Ar*H*), 4.86 (t, 2 H, $^3J_{HH}$ = 6.5 Hz, $N_{Ar}CH_2$), 3.58-3.48 (m, 4 H, OCH_2), 2.94 (t, 2 H, $^3J_{HH}$ = 6.5 Hz, $NC_{Alkyl}H_2$), 2.58-2.48 (m, 4 H, $NC_{ring}H_2$); ^{13}C NMR (400 MHz, CD_3CN): δ_C 153.9, 153.2, 141.8, 141.6, 140.8, 112.5, 111.7, 110.3, 109.6,

106.4, 56.5, 55.6, 54.8, 48.0; ES(+)-MS m/z $C_{25}H_{21}N_2O_3$ Calc. 397.1552, Found 397.1558 a.m.u., Φ=0.042 (10 mM lithium cacodylate buffer (pH 7.3) containing 100 mM potassium chloride).

*1,13-dimethoxy-5,9-bis(2-morpholinoethyl)-5H-quinolino[2,3,4-kl]acridin-9-ium hexaflourophosphate (**3**).* 2-morpholinoethanamine (0.51 mL, 3.95 mmol) was added to a solution of **1** (0.20 g, 0.39 mmol) in 1-methyl-2-pyrrolidinone (1.4 mL). The deep red solution was left to stir at room temperature for 20 min before raising the temperature to 110 °C for 2 h. The deep green solution was then cooled to room temperature and a solution of ammonium hexafluorophosphate (0.13 g, 0.80 mmol) in water (10 mL) was added. The green precipitate formed was recovered by filtration, thoroughly washed with water and then diethyl ether. The solid was dissolved in hydrochloric acid (0.1 M, 30 mL) and washed with dichloromethane (3 × 20 mL). After discarding the organic phase, the aqueous layer was basified with a solution of sodium hydroxide (0.1 M, 60 mL) containing ammonium hexafluorophosphate (0.13 g, 0.80 mmol). The resultant green precipitate was extracted with dichoromethane until the organic phase was a pale blue. The combined organic extracts were dried over magnesium sulphate, filtered and evaporated to dryness *in vacuo*. Repeated re-crystallisation of the solid from acetone / cyclohexane yielded jagged dark green crystals of **3**. Yield: 58 mg, 0.08 mmol, 21%; Elem. Anal. $C_{33}H_{39}N_4O_4(PF_6).H_2O$ Calc. %C 55.15, %H 5.75, %N 7.80, Found %C 55.23, %H 5.42, %N 7.37; ^{1}H NMR (400 MHz, CD_3CN): δ_H 8.19 (t, 1 H, $^3J_{HH}$ = 8.5 Hz, Ar H-7), 7.91 (dd, 2 H, $^3J_{HH}$ = 9.2 and 8.1 Hz, Ar H-3 and H-11), 7.65 (d, 2 H, $^3J_{HH}$ = 8.5 Hz, Ar H-6 and H-8), 7.53 (d, 2 H, $^3J_{HH}$ = 9.1 Hz, Ar H-4 and H-10), 6.93 (d, 2 H, $^3J_{HH}$ = 8.1 Hz, Ar H-2 and H-12), 4.88 – 4.78 (m, 2 H, $N_{Ar}CH_2$), 4.70 – 4.58 (m, 2 H, $N_{Ar}CH_2$), 3.73 (s, 6 H, OCH_3), 3.62 – 3.52 (m, 8 H, $O_{ring}CH_2$), 3.00 - 2.92 (m, 4 H, $NC_{alkyl}H_2$), 2.62 - 2.47 (m, 8 H, $NC_{ring}H_2$); ^{13}C NMR (500 MHz, CD_3CN): δ_C 160.5, 143.9, 143.4, 140.1, 138.0, 137.3, 120.5, 114.2, 108.9, 106.5, 104.0, 67.54, 56.5, 55.3, 54.7, 49.3; ES(+)-MS m/z $C_{33}H_{39}N_4O_4$ Calc. 555.2971, Found 555.2980 a.m.u.

*8,12-bis(2-morpholinoethyl)-8H-benzo[ij]xantheno[1,9,8-cdef][2,7]naphthyridin-12-ium hexafluorophosphate (**DAOTA-M2**).* Pyridine hydrogenchloride (10.00 g) was mixed thoroughly with **3** (0.21 g, 0.30 mmol) and dried under vacuum before the solid was stirred and heated to 200 °C for 1 h with shielding from light. The deep red solution was cooled to room temperature and acetonitrile (100 mL) was added. The crude red solid was recovered by centrifugation at 4,000 rpm for 2 min and washed with acetonitrile (2 × 50 mL) and then diethyl ether (2 × 25 mL). Anion exchange was performed by dissolving the solid in an aqueous solution of sodium hexafluorophosphate (0.1 M, 5 mL), and extracting with dichloromethane (10 mL). The pH of the aqueous layer during this process was adjusted to 14. The addition of an equal volume of ethyl acetate and to organic phase and slow evaporation of this solution when shielded from light gave red rectangular blocks of **DAOTA-M2**. These crystals were dissolved in dichloromethane and the crystallisation process repeated to give **DAOTA-M2**. Yield: 25 mg, 0.04 mmol, 13%; Elem. Anal. $C_{31}H_{33}N_4O_3(PF_6)$

Calc. %C 56.88, %H 5.08, %N 8.56, Found %C 56.49, %H 5.06, %N 8.35%; ^{1}H NMR (400 MHz, CD_3CN): δ_H 8.25 (t, 1 H, $^{3}J_{HH}$ = 8.6 Hz, Ar H-10), 8.10 (dd, 2 H, $^{3}J_{HH}$ = 8.9 and 8.1 Hz, Ar H-2 and H-6), 7.60 (d, 2 H, $^{3}J_{HH}$ = 8.9 Hz, Ar H-1 and H-7), 7.51 (d, 2 H, $^{3}J_{HH}$ = 8.6 Hz, Ar H-9 and H-11), 7.34 (d, 2 H, $^{3}J_{HH}$ = 8.1 Hz, Ar H-3 and H-5), 4.65 (t, 4 H, $^{3}J_{HH}$ = 6.8 Hz, $N_{Ar}CH_2$), 3.64-3.55 (m, 8 H, OCH_2), 2.89 (t, 4 H, $^{3}J_{HH}$ = 6.8 Hz, $NC_{alkyl}H_2$), 2.63-2.53 (m, 8 H, $NC_{ring}H_2$); ^{13}C NMR (400 MHz, CD_3CN): δ_C 153.4, 142.0, 141.0, 140.9, 140.5, 139.4, 112.5, 110.5, 109.4, 108.5, 107.2, 67.5, 54.8, 54.7, 47.2; ES^{+}-MS m/z $C_{31}H_{33}N_4O_3$ Calc. 509.2553, Found 509.2589 a.m.u.; Φ=0.032 (10 mM lithium cacodylate buffer (pH 7.3) containing 100 mM potassium chloride).

Supplementary References

1. Laursen B.W., Krebs F.C. Synthesis, structure, and properties of azatriangulenium salts. *Chem Eur J* **7**, 1773-1783 (2001).

2. Martin J.C., Smith R.G. Factors Influencing the Basicities of Triarylcarbinols. The Synthesis of Sesquixanthydrol. *J Am Chem Soc* **86**, 2252-2256 (1964).
